# A systematic review and meta-analysis reveal that *Campylobacter* spp. and antibiotic resistance are widespread in humans in sub-Saharan Africa

**DOI:** 10.1371/journal.pone.0245951

**Published:** 2021-01-27

**Authors:** Delfina F. Hlashwayo, Betuel Sigaúque, Emília V. Noormahomed, Sónia M. S. Afonso, Inácio M. Mandomando, Custódio G. Bila

**Affiliations:** 1 Department of Biological Sciences, Faculty of Sciences, Eduardo Mondlane University, Maputo, Mozambique; 2 Faculty of Veterinary Science, Eduardo Mondlane University, Maputo, Mozambique; 3 Centro de Investigação em Saúde de Manhiça, Maputo, Mozambique; 4 Department of Microbiology, Faculty of Medicine, Eduardo Mondlane University, Maputo, Mozambique; 5 Infectious Disease Division, Department of Medicine, University of California, San Diego, San Diego, CA, United States of America; 6 Mozambique Institute for Health Education and Research (MIHER), Maputo, Mozambique; 7 Instituto Nacional de Saúde (INS), Ministério da Saúde, Marracuene, Mozambique; Massey University, NEW ZEALAND

## Abstract

**Introduction:**

*Campylobacter* spp. are zoonotic bacteria that cause gastroenteritis in humans worldwide, whose main symptom is diarrhea. In certain cases, extra intestinal manifestations may occur, such as Guillain Barré syndrome. The bacteria cause severe diarrhea mostly in children and in immunocompromised individuals. This review aims to address the prevalence of *Campylobacter* spp. in humans in sub-Saharan Africa. It also aims to understand the impact of HIV in the prevalence, as well as to report data on antibiotic resistance and propose research priorities.

**Methods:**

We followed PRISMA guidelines to find studies on the occurrence of *Campylobacter* spp. in humans in all countries from sub-Saharan Africa. Studies published between 2000 and 2020 were searched in PubMed, Cochrane Library, CINAHL, African Index Medicus, African Journals Online, Google Scholar and Science Direct. We have conducted a random-effect meta-analysis and calculated the proportion of resistant isolates to different antibiotics.

**Results and discussion:**

We found 77 studies that described such occurrence in humans in 20 out of 53 sub-Saharan African countries. *Campylobacter jejuni* was the most prevalent species. Pooled prevalence was 9.9% (CI: 8.4%–11.6%). No major variations within the different sub-regions were found. Most studies reported *Campylobacter* spp. as the cause of diarrhea, mainly in children. Some studies reported the bacteria as a possible etiologic agent of acute flaccid paralysis and urinary tract infection. *Campylobacter* spp. presented a higher pooled prevalence in HIV infected patients, although not statistically significant. High proportions of resistant strains were reported for many antibiotics, including erythromycin and tetracycline.

**Conclusion:**

*Campylobacter* spp. occur in sub-Saharan Africa, although information is scarce or inexistent for many countries. Research priorities should include investigation of the understudied species; extra intestinal manifestations; the impact of HIV infection and associated risk factors. Control strategies should be reinforced to contain the spread of this pathogen and drug resistance.

## Introduction

*Campylobacter* spp. are a group of zoonotic gram-negative bacteria and the leading cause of human bacterial gastroenteritis [1]. In humans, they account for 5%-14% of all diarrheal disease in the world [2]. Cases of *Campylobacter* spp. infection have increased in North America, Europe, and Australia. Some studies indicate that *Campylobacter* spp. infections are also endemic in Africa, Asia, and the Middle East [2–5].

In the United States of America, the incidence of *Campylobacter* spp. infection in 2015 was 12.97 per 100,000 population [6]. In Europe, over 1.8 million *Campylobacter* spp. cases were reported from 2008 to 2016 [7]. The most frequently notified foodborne infection in Australia is from *Campylobacter* spp. with 16,968 notifications in 2010 (112.3 cases per 100,000) [8].

According to a systematic review and meta-analysis published in 2011 [9], *Campylobacter* spp. were identified as some of the most common bacterial gastrointestinal (GI) pathogens in sub-Saharan Africa, with an average of 8.3% in diarrheic and non-diarrheic patients seen in hospitals, primary health care centers or recruited in community cohorts. Other common GI pathogens identified in sub-Saharan Africa were: diarrheagenic *E*. *coli* species, enterotoxigenic *E*. *coli*, *Shigella* spp. and *Salmonella* spp. (30%, 15.4%, 10.5%, 8.4%, respectively) [9].

There are few surveillance systems in place for prevalence data on *Campylobacter* spp. in Africa. However, several reports are published. These bacteria are actually the most commonly reported zoonosis in developed and developing countries [5].

Poor hygiene, lack of adequate sanitation, direct contact between people and contaminated animals, drinking contaminated food and water are some sources of attribution of *Campylobacter* spp. infection in low-income countries [10], most of which are part of sub-Saharan Africa.

*C*. *jejuni* and less frequently, *C*. *coli* cause watery or bloody diarrhea, fever, abdominal cramps and vomiting. Although enteritis caused by these bacteria is sporadic and usually self-limiting, complications such as bacteremia, hepatitis, pancreatitis, lung infections, brain abscesses, meningitis and reactive arthritis can occur [3, 4]. Furthermore, the bacteria have been associated with inflammatory bowel disease and some types of cancer on the intestinal tract [5]. Other *Campylobacter* species have been isolated from humans with disease, such as: *C*. *concisus*, *C*. *fetus*, *C*. *hyointestinalis*, *C*. *insulaenigrae*, *C*. *lari*, *C*. *sputorum*, *C*. *upsaliensis*, *C*. *curvus*, *C*. *gracilis*, *C*. *hominis*, *C*. *rectus*, *C*. *showae* and *C*. *ureolyticus* [3, 11]. Infection by *Campylobacter* spp. is normally limited to children in developing countries [2, 3, 12]. Antibiotics can be considered for treatment in severe cases and in immunocompromised patients [2, 4].

*Campylobacter* spp. are commonly isolated from stools, and less commonly from blood. The optimal conditions for thermophilic *Campylobacter* species growth in culture medium, such as *C*. *jejuni*, *C*. *coli*, *C*. *upsaliensis* and *C*. *lari*, include a microaerophilic environment (5 to 10% of oxygen) and temperature between 40°C and 42°C. Non-thermophilic species (including *C*. *fetus*, *C*. *concisus*, *C*. *curvus*, *C*. *hyointestinalis* and other species) often grow better at 37°C [13, 14]. Another diagnostic test to detect the bacteria is Polymerase Chain Reaction (PCR) [5].

Although much is known about *Campylobacter* spp. in the developed world [15], there are few summarized data about epidemiology, clinical and diagnostic aspects as well as on antibiotic resistance of *Campylobacter* spp. in sub-Saharan Africa [16, 17]. In addition, due to the high prevalence of HIV infection in the region, the impact of this infection in the prevalence of campylobacteriosis is not clearly understood in sub-Saharan Africa.

The available review reports were either circumscribed to one country [18] or restricted to a single *Campylobacter* species [19, 20]. Further, to date, only one meta-analysis on the prevalence of *Campylobacter* spp. in sub-Saharan Africa has been conducted in 2011 [9]. However, updated data are needed. Thus, the aim of this review is to summarize data available regarding human *Campylobacter* spp. prevalence and drug resistance in sub-Saharan Africa from 2000 to 2020, as well as to recommend future research priorities.

## Methods

### Search strategy

A systematic review was conducted according to Preferred Reporting Items for Systematic Reviews and Meta-Analyses (PRISMA) guidelines [21] to summarize available data on human campylobacteriosis from each sub-Saharan African country. The United Nations macro-geographical definition of Africa was used to define the geographical boundaries of this review (https://unstats.un.org/unsd/methodology/m49/), as follows: a) **Eastern Africa**: British Indian Ocean Territory, Burundi, Comoros, Djibouti, Eritrea, Ethiopia, French Southern Territories, Kenya, Madagascar, Malawi, Mauritius, Mayotte, Mozambique, Réunion, Rwanda, Seychelles, Somalia, South Sudan, Uganda, United Republic of Tanzania, Zambia and Zimbabwe; b) **Middle Africa:** Angola, Cameroon, Central African Republic, Chad, Congo, Democratic Republic of the Congo, Equatorial Guinea, Gabon and Sao Tome and Principe; c) **Southern Africa:** Botswana, Eswatini (former Swaziland), Lesotho, Namibia and South Africa; d) **Western Africa:** Benin, Burkina Faso, Cabo Verde, Côte d’Ivoire, Gambia, Ghana, Guinea, Guinea-Bissau, Liberia, Mali, Mauritania, Niger, Nigeria, Saint Helena, Senegal, Sierra Leone and Togo.

A complete study protocol is available in S1 File. PubMed, Cochrane Library, CINAHL, African Index Medicus, African Journals Online, Google Scholar and Science Direct were searched for studies published up to 13 March 2019 without language restrictions. An update of the database search was done on 25 March 2020 using the same set of search terms (detailed search strategy in S2 File). The PRISMA checklist is available in S1 Table.

Selection criteria of studies included:

- The study population consisted of any group of people of all age groups in sub-Saharan Africa who had been tested for *Campylobacter* spp.;
- Descriptive, cross-sectional studies, prospective, or retrospective studies and case reports and series in which the prevalence of *Campylobacter* spp. in any country in sub-Saharan Africa was reported;
- Conference abstracts and
- Only studies published since 2000.

The main exclusion criteria of studies included:

- Review articles;
- Countries not belonging to sub-Saharan Africa;
- Experimental data.

The primary outcome in the review was the prevalence of *Campylobacter* spp. in countries from sub-Saharan Africa. The main secondary outcomes were the antibiotic resistance proportions.

### Risk of bias (quality) assessment

Risk of bias was assessed separately for each eligible study, using an assessment tool with slight modifications [22]. Details of this assessment can be found in S2 Table. No study was excluded based on its quality.

### Data extraction (selection and coding)

Titles and abstracts were screened for location, study population and general correlation with the research objectives. Full versions of potentially relevant articles were obtained to assess eligibility. Two researchers (DFH and SMSA) screened independently studies and determined study eligibility. Cross-references of the full text retrieved articles were also searched. Data were collected independently from each publication and captured using a standardized Word document form. Data were extracted from text, tables and figures.

### Data analysis

In reports where the numerator and denominator of the study sample were available, prevalence data were calculated, if not already provided. When not presented in the manuscript, the 95% exact confidence intervals (CI) were calculated, using the “binom.test” function (“stats” package) in R 3.5.1.

A meta-analysis was performed using MetaXL version 5.3 (https://www.epigear.com/index_files/metaxl.html) (EpiGear International Pty Ltd, Australia). The software was used to produce the pooled estimates, forest plots, as well as to calculate the Cochran’s Q and p values. Study heterogeneity (Cochran’s Q) was evaluated by I^2^ (level of inconsistency) [23]. The I^2^ values above 75% were considered as a high degree of heterogeneity [24].

The pooled prevalence estimates of *Campylobacter* spp. infection and 95% CIs were calculated assuming a random-effect model. Subgroup analysis were conducted to sub-Saharan African regions; study subjects (patients with and without diarrhea; HIV infected and uninfected patients); years of publication and age. A separate pooled prevalence analysis was made to each *Campylobacter* species.

Regarding antibiotic resistance, the proportions of resistant *Campylobacter* spp. isolates were calculated dividing the number of resistant isolates by the number of tested isolates.

## Results

### Search results

**Fig 1:**
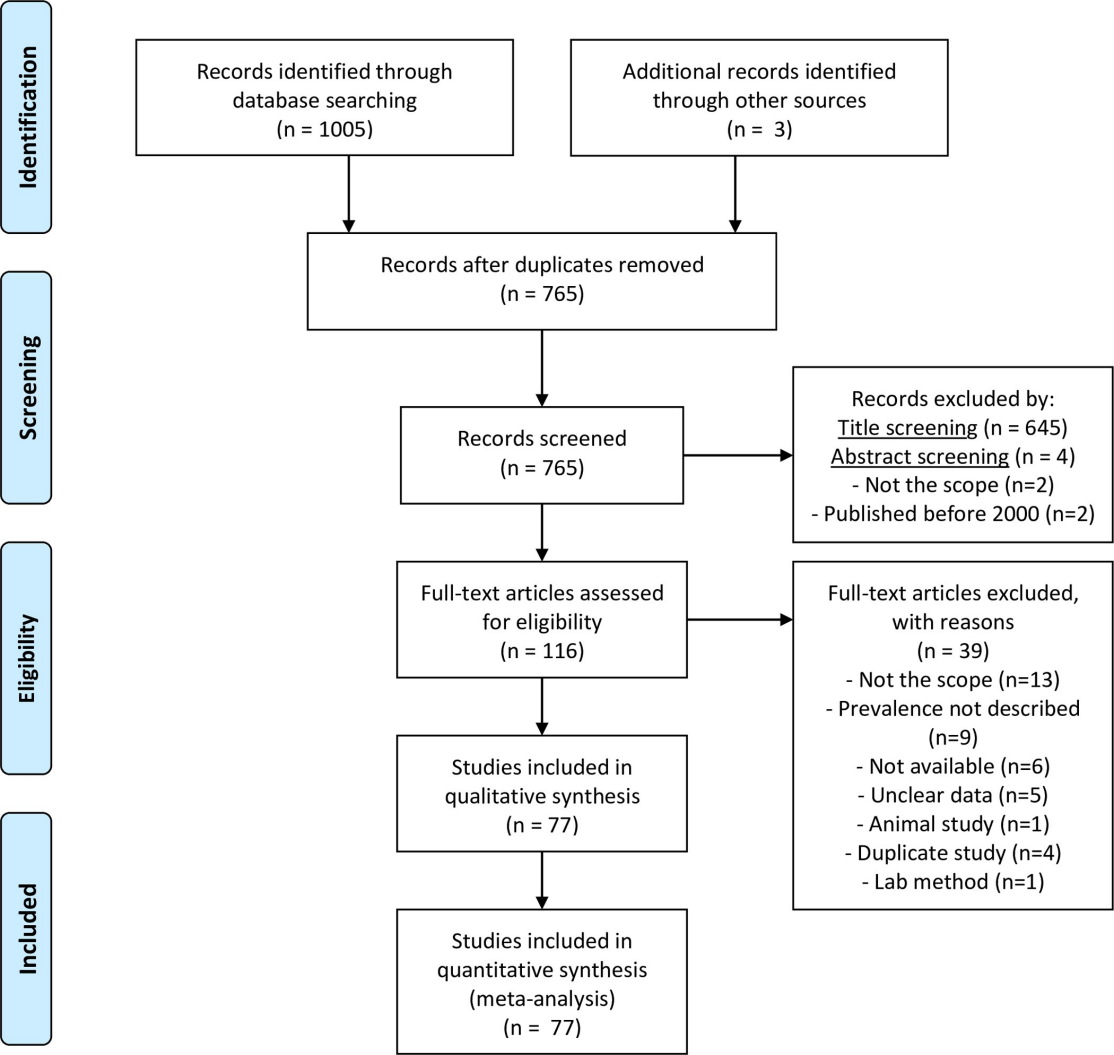
PRISMA flow diagram of study selection.

Results from the search are summarized in Fig 1. Most records were published in English, except for one that was published in French. From the 77 total records [25–101], 70 were research articles, 1 was a PhD thesis, 3 were MSc dissertations and 3 were conference abstracts.

The studies were originated from 20 out of 53 sub-Saharan African countries, namely: Angola, Botswana, Burkina Faso, Côte d’Ivoire, Ethiopia, Ghana, Guinea Bissau, Kenya, Madagascar, Malawi, Mozambique, Niger, Nigeria, Rwanda, Senegal, South Africa, Tanzania, Uganda, Zambia and Zimbabwe. Overall quality assessment scores for risk of bias in studies included in the review ranged from three to ten. Of the total 77 studies assessed, one study had a score of 3, fifty were scored >7, of which twenty-five were scored >8. Twenty-six studies had a score between 4 and 7.

The majority of studies were based in Kenya (n = 13), Nigeria (n = 12), Tanzania (n = 9), South Africa (n = 8) and Ethiopia (n = 7). The number of samples per study ranged from 40 to 5635. Of the total 77 studies, 33 were recorded in the past 10 years (since 2010) and 6 studies did not report about data collection time frame. Prevalence ranged from 0% to 68%. Nine different *Campylobacter* spp. were identified, namely: *C*. *jejuni*, *C*. *coli*, *C*. *lari*, *C*. *hyointestinalis*, *C*. *fetus*, *C*. *curvus*, *C*. *sputorum*, *C*. *concisus* and *C*. *upsaliensis*. Nevertheless, *Campylobacter* species were not identified to species level in thirty studies. Table 1 provides an overview of the included studies.

**Table 1:** Overview of the studies included in the review.

	N (%)
**sub-Saharan Africa region**	
Eastern Africa	43 (55.8%)
Middle Africa	1 (1.3%)
Southern Africa	11 (14.3%)
Western Africa	22 (28.6%)
**Type of samples**	
Feces	75 (97.4%)
Urine and feces	1 (1.3%)
Blood and feces	1 (1.3%)
**Setting**	
Hospitals	66 (85.7%)
Communities, farms or peri urban areas	6 (7.8%)
Others^a^	4 (5.2%)
Only primary schools	1 (1.3%)
**Type of study**	
Cross-sectional	49 (63.6%)
Case-control	20 (26.0%)
Cohort	7 (9.1%)
Prospective	1 (1.3%)
**Human subjects age**	
Children below 5 years	39 (50.6%)
Children aged 10–15 years	9 (11.7%)
Above 18 years	2 (2.6%)
Random ages	27 (35.1%)
**Diagnostic methods**	
Routine culture only	12 (15.6%)
Routine culture and other methods	47 (61.0%)
PCR only	16 (20.8%)
Not reported/ not clear	2 (2.6%)
**Type of subjects**	
With diarrhea	34 (44.1%)
With or without diarrhea	26 (33.8%)
Asymptomatic	2 (2.6%)
Others^b^	15 (19.5%)

N- Number of human studies.

^a^ One study was based on laboratory records, one other was unclear and two studies were from both schools and hospitals;

^b^ These included: malnourished patients; volunteers; patients with and without enteric complaints; patients with acute flaccid paralysis; patients with urinary tract infection and HIV infected patients.

S3 Table presents detailed information about *Campylobacter* spp. prevalence in studies found in sub-Saharan Africa. There were no outbreak reports.

### Pooling estimates of *Campylobacter* spp. in humans

**Fig 2:**
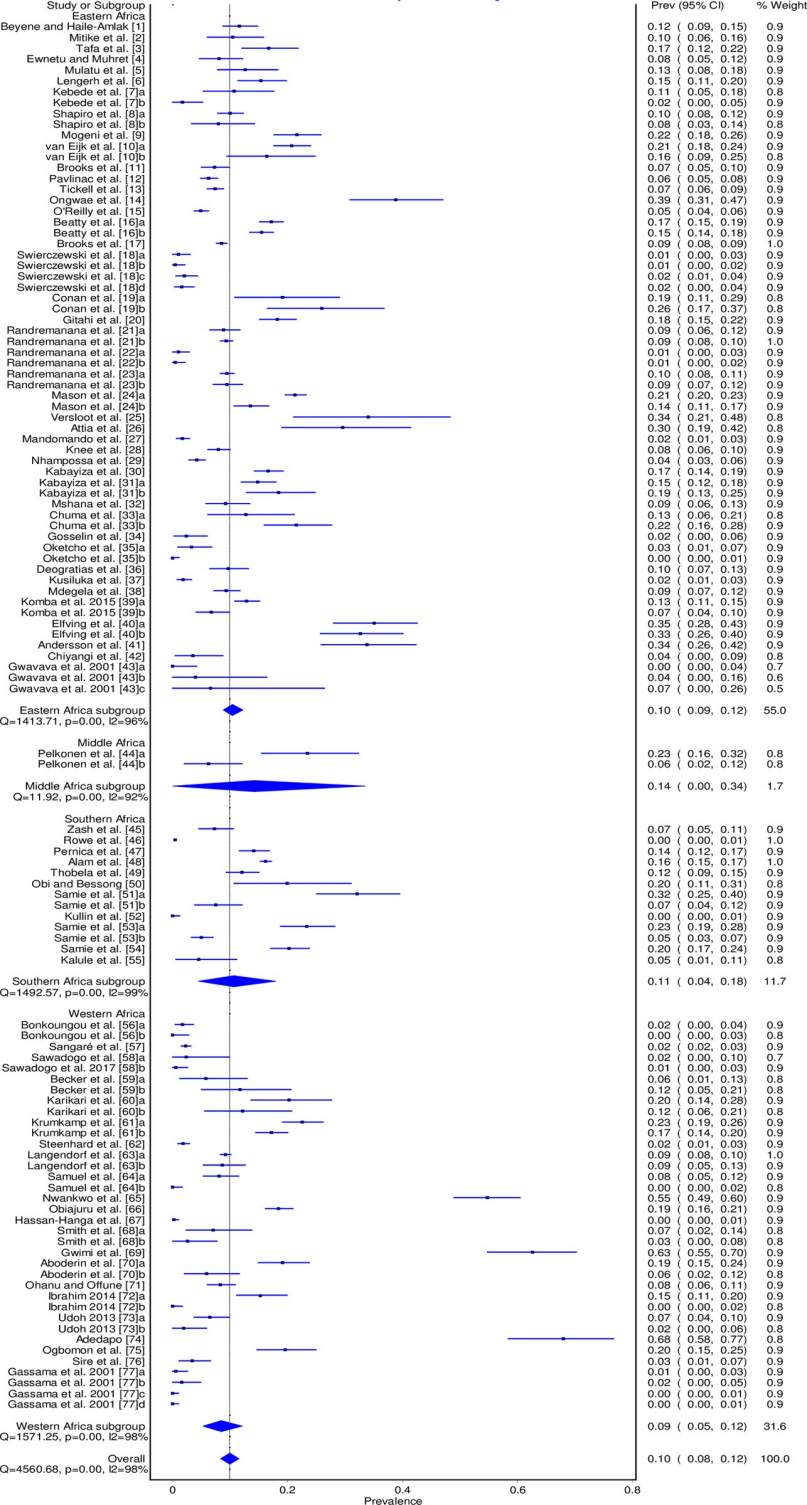
Forest plot of *Campylobacter* spp. pooled prevalence in humans in sub-Saharan Africa between 2000 and 2020.

The pooled prevalence estimates of *Campylobacter* spp. in humans with individual studies are shown in a forest plot (Fig 2). We have excluded data on the control group from Nhampossa et al. 2015 [46] study because they were not clear. Overall, studies from sub-Saharan Africa revealed a pooled prevalence of 9.9% (95% CI: 8.4%–11.6%). There was a substantial heterogeneity among studies (I^2^ = 98%; p = 0.00).

The summary of the subgroup analysis is found in Table 2. The prevalence among the sub-regions varied between 8.5% (95% CI: 5.3% - 12.1%) and 14.1% (95% CI: 0.0% - 33.6%).

**Table 2:** Pooled prevalence of *Campylobacter* spp. stratified by subgroups.

	Sample size	No. positive	No. of studies	Pooled Prev.	95% CI	Cochran’s Q	Heterogeneity I^2^ (%)	p value	Weight (%)
**Overall**	59,249	6,519	77	**9.9**	8.4–11.6	4,560.7	98	0.00	100
**Region**									
Eastern Africa	32,256	3,614	43	**10.5**	8.8–12.2	1,413.7	96	0.00	55.0
Middle Africa	194	29	1	**14.1**	0.0–33.6	11.9	92	0.00	1.7
Southern Africa	13,578	1,409	11	**10.7**	4.5–17.9	1,492.6	99	0.00	11.7
Western Africa	13,221	1,467	22	**8.5**	5.3–12.1	1,571.3	98	0.00	31.6
**Patients with or without diarrhea**^a^									
With diarrhea	32,965	3,749	60	**10.2**	8.6–11.9	1,473.3	96	0.00	68.9
Without diarrhea	9,143	939	28	**6.5**	4.1–9.2	635,1	95	0.00	31.1
**HIV positive or negative**									
HIV positive	1,387	190	13	**10.8**	3.9–19.2	331.6	95	0.00	56.2
HIV negative	5,273	413	9	**8.4**	4.6–13.0	277.8	96	0.00	43.8
**HIV positive patients: With or without diarrhea**									
HIV with diarrhea	916	115	11	**10.7**	5.1–17.6	85.5	88	0.00	71.7
HIV without diarrhea	364	4	5	**1.4**	0.1–3.2	5.6	29	0.23	28.3
**Years**									
2000–2005	3,644	278	10	**5.5**	3.0–8.5	206.3	92	0.00	15.0
2006–2010	22,169	2,260	16	**9.2**	5.5–13.4	1,986.6	99	0.00	19.3
2011–2015	27,026	3,049	29	**9.5**	7.5–11.7	1,432.5	97	0.00	40.8
2016–2020	6,410	932	22	**14.7**	9.9–19.9	866.8	97	0.00	24.9
**Age**									
Below 15 years	39,256	4,768	48	**10.5**	8.9–12.2	1,768.7	96	0.00	61.0
Above 18 years	295	15	2	**4.6**	0.6–9.8	12.1	67	0.02	3.6
Random ages^b^	19,698	1,736	27	**9.5**	6.2–13.1	2,405.1	98	0.00	35.4
**Study setting**									
Communities	7,884	759	6	**11.7**	7.3–16.5	304.7	97	0.00	8.3
Hospitals	48,968	5,422	66	**9.5**	7.8–11.4	4,089.7	98	0.00	84.6
Primary schools	580	106	1	**18.3**	15.2–21.5	-	-	-	0.9
Others	1,817	232	4	**11.8**	5.4–19.1	115.6	95	0.00	6.2
***Campylobacter* spp.**									
*C*. *jejuni*	38,775	3,136	46	**7.6**	6.2–9.0	1,463.3	96	0.00	100
*C*. *coli*	28,343	632	32	**3.4**	2.4–4.4	714.2	95	0.00	100
*C*. *lari*	8,401	115	10	**3.0**	0.7–6.8	358.7	97	0.00	100
*C*. *hyointestinalis*	6,015	21	3	**1.3**	0.0–3.6	16.4	88	0.00	100
*C*. *fetus*	6,123	24	4	**2.8**	0.0–8.9	83.9	96	0.00	100
*C*. *curvus*	5,635	1	1	**0.02**	0.0–0.1	-	-	-	100
*C*. *sputorum*	5,635	1	1	**0.02**	0.0–0.1	-	-	-	100
*C*. *upsaliensis*	7,131	266	4	**5.6**	0.6–14.2	189.9	98	0.00	100
*C*. *concisus*	5,957	244	2	**4.1**	3.6–4.6	0.9	0	0.64	100

^a^ Number of studies is greater than 77 because 26 studies included both groups with and without diarrhea.

^b^ Random ages refers to studies that included patients from a wide range of ages and to 3 studies where age-related data were not reported.

No statistically significant differences were found in *Campylobacter* spp. prevalence in patients with and without diarrhea, as well as between HIV infected patients and HIV uninfected ones. When comparing HIV-infected patients, *Campylobacter* spp. prevalence was significantly higher in patients with diarrhea when compared to non-diarrheic HIV patients (10.7% (95% CI: 5.1% - 17.6%) and 1.4% (95% CI: 0.1% - 3.2%)), respectively. There was an increase in *Campylobacter* spp. pooled prevalence over time and a higher prevalence was found in children under 15 years, although not statistically significant as well.

*C*. *jejuni* was the most tested species and with the highest pooled prevalence (7.6%). Table 3 presents the pooled prevalence of *Campylobacter* species with subgroup analysis by the presence of diarrhea and by sub-Saharan African region. The table does not include *C*. *curvus* and *C*. *sputorum* because these species presented the lowest prevalence (0.02% for both) and were tested in the same study from South Africa [67].

**Table 3:** Pooled prevalence and 95% CI of different *Campylobacter* species according to presence of diarrhea and sub-Saharan African region.

	*C*. *jejuni*	W	*C*. *coli*	W	*C*. *lari*	W	*C*. *hyointestinalis*	W	*C*. *fetus*	W	*C*. *upsaliensis*	W	*C*. *concisus*	W
**Patients with or without diarrhea**														
With	8.3 (6.3–10.4)	76.4	2.5 (1.7–3.6)	72.4	0.9 (0.5–1.5)	71.9	1.3 (0.2–3.3)	100	1.7 (0.1–4.6)	100	-	-	3.8 (1.2–7.3)	50.3
Without	4.9 (1.4–9.3)	23.6	2.6 (1.1–4.3)	27.6	2.0 (0.9–3.2)	28.1	-	-	-	-	-	-	2.6 (0.5–5.6)	49.7
**sub-Saharan African region**														
EA	7.5 (5.6–9.6)	47.8	2.2 (1.5–3.0)	45.6	1.3 (0.8–1.9)	37.3	-	-	-		-	-	-	-
MA	-	-	-	-	-		-	-	-		-	-	-	-
SA	9.9 (6.0–14.2)	11.9	3.5 (1.1–6.5)	15.2	0.0 (0.0–0.1)	9.7	0.2 (0.1–0.4)	38.7	0.0 (0.0–0.1)	26.4	3.2 (3.5–4.5)	26.3	4.1 (3.6–4.6)	100
WA	6.9 (4.3–9.8)	40.3	4.7 (1.6–8.5)	39.2	6.5 (0.2–16.0)	53.0	2.0 (0.5–4.6)	61.3	3.9 (0.0–12.9)	73.6	7.1 (0.0–29.7)	73.7	-	-

EA–Eastern Africa; MA–Middle Africa; SA–Southern Africa; WA: Western Africa; W—Weight

### Resistance of *Campylobacter* spp. isolates to antibiotics

From the 77 studies included in this systematic review, a total of 31 reported antibiotic resistance [25, 30, 32, 33, 36, 37, 44, 48, 50, 57, 59, 61, 64, 65, 69, 70, 73–75, 77, 80, 81, 84, 85, 87, 89, 91, 94–96, 99]. Data were from Eastern, Southern and Western Africa. S4 Table provides detailed information for antibiotic resistance within each study.

Antibiotic resistance was screened for *C*. *jejuni* (916 isolates), *C*. *coli* (154), *C*. *lari* (44), *C*. *upsaliensis* (33), *C*. *fetus* (16) and other *Campylobacter* spp. not identified to species level (534). Isolates were tested to a total of 48 different antibiotics. Table 4 presents the summary of *Campylobacter* spp. resistance to the 5 most tested antibiotics.

**Table 4:** Summary of human *Campylobacter* spp. resistant isolates to the most tested antibiotics.

Region	Proportion % (number of resistant isolates)				
	Erythromycin	Tetracycline	Ciprofloxacin	Nalidixic acid	Gentamicin
**Eastern Africa**	54% (335/625)	44% (267/605)	21% (122/575)	36% (199/555)	39% (119/304)
**Southern Africa**	70% (157/225)	30% (68/225)	12% (30/254)	52% (116/225)	19% (42/225)
**Western Africa**	13% (65/512)	49% (172/353)	11% (34/304)	24% (66/279)	24% (194/490)
**sub-Saharan Africa**	**41% (557/1,362)**	**43% (507/1,183)**	**16% (186/1,133)**	**36% (381/1,059)**	**35% (355/1,019)**

The highest proportion of resistant isolates among the 10 most tested antibiotics (S4 Table), was for ampicillin (63%), trimethoprim sulfamethoxazole (49%), tetracycline (43%) and erythromycin (41%). However, many other antibiotics presented a high proportion of resistant isolates as presented in S4 Table.

Multidrug resistance, referred as resistance to more than two drugs, was reported in a total of seven studies [36, 37, 57, 59, 70, 80, 84].

## Discussion

To our best knowledge, this is the most recent systematic review and meta-analysis that summarized and analyzed data about the prevalence, distribution and antibiotic resistance of *Campylobacter* species in sub-Saharan Africa.

We found a pooled prevalence of *Campylobacter* spp. in humans of 9.9%, with a greater representation of patients with diarrhea. This prevalence is close to an average prevalence of 8.3% previously reported in the region [9].

Most of the reports have focused on diarrhea-related comorbidities, although the differences in the prevalence of *Campylobacter* spp. in patients with and without diarrhea were not statistically significant.

It is understandable that most of the studies were based in Eastern Africa (55.8%) that has the majority of countries of sub-Saharan African region (22 countries, 41.5%). These data are consistent with the sample size because Eastern Africa presented the largest sample size and weight (n = 32,256, weight = 55.0%). Middle Africa, with the lowest weight in the meta-analysis (1.7%) is the second region with the less countries in sub-Saharan Africa (9/53 countries). Thus, there was a low representativeness of this region in this review.

In Eastern Africa, Kenya and Tanzania had most studies (13 and 9, respectively). Accordingly, Kenya had the highest percentage of samples analyzed in the sub-region, followed by Madagascar (42%, and 20%, respectively). However, Tanzania had on average fewer samples analyzed per publication than Madagascar, but the later had a greater weight in the meta-analysis.

The presence of *Campylobacter* spp. in asymptomatic individuals [34–36, 38–41, 49, 51, 53, 58, 63, 71, 73, 76, 78, 79, 81, 82, 85, 89, 91, 92, 94, 95, 99–101] is noteworthy, both due to the considerable number of reports, as well as because it appears to occur frequently in sub-Saharan Africa. It may result from continued exposure probably at low doses [40]. This exposure may be due to poor hygienic-sanitary conditions [36, 38, 41, 58], including contact with reservoir animals such as chickens [36], and has probably resulted in developed immunity against the pathogen [38, 40, 41]. In certain cases, the asymptomatic carriage could be consequence of a resolved infection in which the bacteria continues to be eliminated in the feces [41, 79].

*Campylobacter* spp. are known to be excreted for up to 12 weeks and even to 40 weeks after infection [102]. Considering the common sanitary conditions in sub-Saharan Africa, it is more likely that the asymptomatic carriage is a result of developed protective immunity [4], meanwhile, when the individual is exposed to high doses of the bacteria, a symptomatic infection may occur [40]. Although there is an asymptomatic population, *Campylobacter* spp. have been proven to cause diarrhea in some of the studies included in this review [25, 41, 53, 63, 71, 73, 81, 85, 91, 94].

The findings of this study demonstrated no statistically significant differences in the prevalence of *Campylobacter* spp. in both HIV positive and negative patients. However, when analyzing particularly HIV infected patients, the ones with diarrhea presented a higher prevalence of *Campylobacter* spp. when compared to HIV patients without diarrhea. In view of this, *Campylobacter* spp. may be predominantly symptomatic in HIV-positive patients; and this can be supported by previous reports that showed the association of HIV with the prevalence and/or severity of campylobacteriosis [77, 101, 103–105]. This needs to be clarified, especially for the sub-Saharan African context, as it bears 66% of new HIV infections worldwide [106]. Thus, in order to understand the impact of HIV in campylobacteriosis, future studies should include HIV serological status data, including CD4 cell count and/or viral load and if possible the intake or not of highly active antiretroviral treatment (HAART).

The increasing prevalence of *Campylobacter* spp. over time in sub-Saharan Africa is in concordance with the global data [3, 4]. These findings can be due to the expanded use of molecular techniques for *Campylobacter* spp. detection or because more susceptible patients were studied. Regardless of these assumptions, this increased prevalence should raise the concern of health authorities in regard to these bacteria.

The pooled prevalence among the sub-regions suggests that the pathogen has a nearly uniform epidemiology in the sub-Saharan region, which can be explained by the similar hygienic-sanitary conditions. Fact remains that Middle Africa revealed the highest pooled prevalence (14.1%) most likely because only one study contributed with a few amount of data. The lowest prevalence found in Western Africa, although not quite different from other regions, may be the result of some control measures in place aiming at hygiene promotion and improving sanitation in rural and urban areas, with a main contribution from Senegal and Burkina Faso [107, 108], as well as from Guinea-Bissau, Côte d’Ivoire, and Niger [109].

The high pooled prevalence of *Campylobacter* spp. in children, particularly those under 15 years is also in agreement with the findings worldwide. It is known that children, mainly the ones under 5 years are the most affected by diarrhea caused by *Campylobacter* spp. [2, 3]. In the meta-analysis this group presented a greatest weight (61.0%), but it was difficult to compare to other age categories due to the uneven way of reporting data.

Despite the fact that the majority of studies tested *Campylobacter* spp. occurrence in stool as cause of diarrhea, one study tested that type of sample with the purpose of knowing whether these bacteria were the cause of acute flaccid paralysis [68]. Analysis of samples other than stool are important, although they were few in the region, particularly for urine [80] and blood [62]. Despite these few data, it was noticed that the bacteria were present in patients diagnosed with Urinary Tract Infection (UTI) [80]. Further research is needed to identify the presence of *Campylobacter* spp. in diseases or symptoms other than gastroenteritis, including bacteremia and UTI. These studies would contribute to identify the tissues or organs to which the bacteria often spreads, along with the clinical presentation.

The substantial heterogeneity among studies (I^2^ = 98%) can be explained by the different types of studies, settings (rural, urban, hospitals), inclusion criteria, sample size, detection methods, data collection time-period and identified species.

It was to be expected that the majority of studies were hospital-based (85.7%), considering that these are less costly, easier and faster to perform. However, studies at community level are important to comprehensively understand *Campylobacter* spp. association with different factors related to environment, hygiene practices, presence of animals, and to analyze in more detail the presence of the pathogen in symptomatic and asymptomatic groups.

The fact that a higher prevalence was detected in schools and communities can be possibly explained by the poor hygiene conditions in those settings, which contribute to human contamination. Nevertheless, these studies presented a small amount of data when compared to studies carried out in hospitals and the difference was not statistically significant.

Notwithstanding the fact that cross-sectional and case-control studies provide useful epidemiological information, cohort studies provide the strongest scientific evidence, but they were less frequent in sub-Saharan African region (9.1%) when compared to cross-sectional (63.6%) and case-control (26.0%). Accordingly, cohort and case-control tested fewer samples when compared to cross-sectional studies (6,200, 13,313 and 39,336) respectively.

The identification of *Campylobacter* spp. through culture was the most common (15.6% and 9,489 samples, in addition to culture coupled with other methods (61.0% and 40,436 samples)), when compared with other PCR alone (20.8% and 8,570 samples). Culture method has limitations because *Campylobacter* spp. can become unviable during transport and processing. They can also survive as viable but non culturable forms that will not grow on selective media [110]. These facts can lead to false-negative results. Besides that, certain culture methods cannot isolate a wide variety of species. For example, *Campylobacter ureolyticus* cannot be detected on Preston agar under microaerobic conditions at 42°C [111]. Selective culture media containing antibiotics, the common method for culturing *Campylobacter* spp. from feces can be unable to detect some species including *C*. *hyointestinalis*, *C*. *fetus* and *C*. *upsaliensis*, which are sensitive to antibiotics used in these media [112]. Probably for these reasons there was a misrepresentation of *Campylobacter* species other than *C*. *jejuni* and *C*. *coli*.

Culture is a more time-consuming method and with fewer sensitivity [113], compared to modern molecular methods such as PCR. However, PCR results must also be analyzed with caution, as the method can detect fragments of DNA that may not correspond to a current infection [63].

*C*. *jejuni* was the most searched and reported species. It presented the highest prevalence mainly in patients with diarrhea when comparing with other *Campylobacter* spp. Nonetheless, *C*. *coli* also exhibited a notable prevalence (3.4%) that was approximate from the other species such as *C*. *upsaliensis*, *C*. *concisus*, and *C*. *lari* (5.6%, 4.1% and 3.0%, respectively). These data must be interpreted cautiously in view of the fact that these three species were tested in a fewer amount of samples. Moreover, *C*. *curvus* and *C*. *sputorum* were less commonly searched and reported [67].

The contribution of *C*. *coli*, *C*. *lari* and *C*. *concisus* for diarrhea in humans in sub-Saharan Africa does not appear to be prominent as the differences in prevalence in case and control groups was not statistically significant. The role of other *Campylobacter* species on the onset of diarrhea is not clear, since it was not possible to compare diarrheic and non-diarrheic groups, although they were often isolated in patients with gastrointestinal complaints.

Although 33 out of 53 sub-Saharan African countries had no data, it is very likely that *Campylobacter* species occur there, given the fact that these bacteria are found in the nearby countries in the four sub-regions.

In general, some studies demonstrated that *Campylobacter* spp. prevalence can be worsened due to other comorbidities such as malnutrition [42, 43, 98], HIV/AIDS [77, 89], malaria [54], giardiasis [82], and, rotavirus and norovirus infection in children [41]. In some studies, *Campylobacter* spp. prevalence was clearly associated with a younger age [33, 38, 40, 56, 57].

Risk factors that may have contributed to the bacteria prevalence were identified in some studies, and included: living in densely populated localities [51]; consumption of chicken meat [57, 94], pre-prepared salad [57], raw food [81]; canned milk [94]; drinking contaminated water [70, 94, 114]; inadequate hygienic-sanitary conditions [27, 35, 45, 51, 90, 95]; malnourishment [70]; contact with persons with diarrhea in the household [26]; and contact with animals in households [37, 77, 95] such as cats [25, 26, 70], dogs [25, 70], chickens [70, 94], goats [35], sheep [94], cattle [55], pigeons [70] and pigs [90].

The antibiotic resistance is a matter of concern due to the greater risk for treatment failure [80]. The antibiotic resistance of *Campylobacter* spp. isolated from animals in sub-Saharan Africa has already been reported [115]. This is of great concern due to frequent contact with animals that can transfer resistance genes to human *Campylobacter* spp. isolates and worsen the situation [116]. Furthermore, humans can acquire pathogens with resistance directly from animals and animal products [117, 118].

This review found resistance to antibiotics used for treatment of campylobacteriosis, including erythromycin (41%) which is considered the drug of choice for clinical treatment of campylobacteriosis [119], as well as to tetracycline and gentamicin (43% and 35%) which are used in treatment of systemic infections [119]. Ciprofloxacin resistance proportion (16%), although considerable and reported globally [120], was not as prominent as of other antibiotics. Ciprofloxacin is also among the clinical used drugs for treatment of campylobacteriosis [119]. Among the most tested antibiotics, ampicillin revealed to have a higher resistance proportion (63%) (S4 Table). The resistant scenario is thought to be the result of indiscriminate use both by humans and in animal husbandry [57, 89].

In general, *Campylobacter* spp. may not have been so extensively studied in all countries from sub-Saharan Africa because they are not part of routine lab investigations for etiology of diarrhea in the countries such as Burkina Faso [76]. Some studies screened for other enteric pathogens such as *Escherichia coli* and *Salmonella* spp. in addition to *Campylobacter* spp. In some of these studies *Campylobacter* spp. where the most frequent bacterial pathogens [25, 26, 32, 40, 59, 64, 69, 73, 81, 87, 101], while not in other studies [30, 31, 61, 62, 65, 76, 88, 100].

Taking into consideration the findings of this systematic review and meta-analysis, we outline below research priorities for future *Campylobacter* spp. studies in sub-Saharan Africa. First we propose conducting of clinical, epidemiological and molecular studies, reflecting different seasonal variations and risk factors for infection. Studies should include antibiotic resistance data and search for antibiotics alternatives, as well as for molecules that can be candidates for new drugs [121]. We suggest the use of molecular techniques in replacement or in combination with culture, in order to provide the real burden of *Campylobacter* spp. infection and to characterize the genetic variants that occur in the region since these variations affect antibiotic resistance and pathogenicity [122]. Point of care diagnosis should be developed as well. Research questions should be broadened to find out the role of *Campylobacter* spp. in diseases other-than diarrhea, as well as to identify *Campylobacter* spp. other than *C*. *jejuni* and *C*. *coli*. Special attention should be given to conduct HIV-related research, specifically to analyze whether there is a relationship with the severity of disease caused by *Campylobacter* spp. and the degree of immunosuppression or viral load. It would also be important to know if HIV patients are at more risk for a bloodstream infection than HIV uninfected patients; and whether HAART therapy has a protective effect in campylobacteriosis.

Although we have systematized data concerning the occurrence of *Campylobacter* spp. and their resistance to antibiotics, this study presents the following limitations: a comparison of gender was not performed due to insufficient data in many studies; it was not possible to compare the prevalence in both urban and rural areas for the same reason; the age subgroup comparison was not sufficiently comprehensive due to heterogeneous and unreported data; antibiotics resistance data may have not covered all published studies in the region because the selected studies had to include prevalence data (that was the primary outcome of this review); and the last point is that it was not possible to compare resistance patterns by the different species due to the inconsistent data reported within each study.

## Conclusions

*Campylobacter* spp. occur in humans in sub-Saharan Africa and presented a pooled prevalence of 9.9% (8.4%– 11.6%). No major prevalence variations were found within the sub-regions. These bacteria prevail mainly as an important causative agent of gastrointestinal disorders, particularly diarrhea. In addition, extra-intestinal infections such as acute flaccid paralysis and urinary tract infection occur, although data are not widely comprehensive.

Of the total nine isolated species, the one with broadest epidemiological understanding is *C jejuni*. Species like *C*. *curvus*, *C*. *sputorum* and *C*. *concisus* are understudied, thus deserve attention in further research, as they may be important in the etiology of diarrhea. HIV infection may contribute to the onset of campylobacteriosis in patients, since patients with HIV are often symptomatic when infected with *Campylobacter* spp.

Resistance to antibiotics is a matter of concern in the region, which may be due to the indiscriminate use both in the treatment of diarrhea and in animal husbandry. Consequently cross-contamination occurs, which increases the resistance proportion.

As research priorities, we propose the implementation of surveillance systems for common *Campylobacter* spp. in order to quantify the disease burden; obtaining data from the countries where they were not available from, and to study the less common species. The clinical presentation, including the prevalence of extra intestinal complications and risk factors for campylobacteriosis should also be addressed in future studies. Considering the high prevalence of HIV in the continent, epidemiological studies of *Campylobacter* spp. should compare the prevalence and severity of disease in both HIV positive and negative patients, as well as the influence of the viral load, CD4 cell count and HAART therapy. Finally we recommend the search for molecules that can be candidates for new antibiotics and to characterize the different *Campylobacter* spp. genotypes existent in the sub-Saharan African region.

## Supporting information

S1 File Protocol for the systematic review. (DOCX)

S2 File Search strategy. (DOCX)

S1 Table PRISMA 2009 checklist. (DOC)

S2 Table Risk of bias (quality) assessment for included human studies. (DOC)

S3 Table Detailed information about *Campylobacter* spp. studies in humans from sub-Saharan Africa from 2000 to 2020. (DOCX)

S4 Table Summary of *Campylobacter* spp. antibiotic resistance data of sub-Saharan Africa. (XLSX)
