# Supplementary material for: A systematic review and meta-analysis reveal that Campylobacter spp. and antibiotic resistance are widespread in humans in sub-Saharan Africa
Source: PLoS One. 2021 Jan 27;16(1):e0245951. doi: 10.1371/journal.pone.0245951 (PMC7840040; doi:10.1371/journal.pone.0245951)
Supplement: S1 File — (DOCX) [file pone.0245951.s001.docx]

**A systematic review and meta-analysis reveal that *Campylobacter* spp. and antibiotic resistance are widespread in humans in sub-Saharan Africa**

| Protocol for systematic review |
| --- |

**Background**

*Campylobacter* spp. are zoonotic pathogens with a worldwide distribution. They can contaminate animals such as chickens, cattle, sheep, and pigs and can also be found in birds, reptiles and shellfish. The bacteria are gram-negative, non-spore forming and exist as either curved or spiral shaped rods [1]. *Campylobacter jejuni* and *C. coli* are recognized to be common human enteric pathogens. *C. jejuni* is a leading cause of gastroenteritis worldwide [2]. In developing countries, *Campylobacter* spp. are a significant bacterial cause of diarrhea in children [3]. Although there are indications of the presence of the bacteria in humans in sub-Saharan Africa, it is lacking an extensive overview of its distribution and epidemiology in this region. The aim of the review is to summarize the data for campylobacteriosis in humans available from sub-Saharan Africa, and to best describe present knowledge of the epidemiology of this infectious disease in the region.

**Objectives**

To summarize the data available for *Campylobacter* spp. in humans in sub-Saharan Africa^[[1]](#footnote-1)^, which includes the epidemiology of human campylobacteriosis infection, outbreaks, clinical manifestations, diagnostics, antibiotic resistance, control and prevention measures and recommendations for further research.

**Outcome(s)**

- *Primary outcomes*

The primary objective is to investigate the prevalence (and/or incidence) of campylobacteriosis in humans in all countries of sub-Saharan Africa.

- *Secondary outcomes*

Secondary objectives include risk factors, circulating strains, clinical manifestations, treatment, prevention measures, mortality and antibiotic resistance.

**Identifying research evidence**

We will conduct a systematic review according to the PRISMA guidelines to summarize the data for campylobacteriosis available from each country of sub-Saharan Africa. We will search Cochrane Library, CINAHL, African Index Medicus, African Journals Online, Google Scholar, PubMed (non-Medline citations) and Science Direct for studies published up to 13 March 2019 without language restrictions. If necessary, we will update the search before submission. Any study in which the epidemiology of campylobacteriosis in any country in sub-Saharan Africa is reported will be included, as well as case series and case reports. Experimental microbiological studies will be excluded.

Title and abstract review exclusion criteria (with coding hierarchy)

1. Article type
   1. Review or summary article without original data
   2. Editorial, letter to the editor opinion, commentary or policy article without original data
   3. Textbook or handbook rather than publication of new data
   4. Lay media publications or broadcasts
2. Geographic focus: Exclude countries out-with sub-Saharan Africa
3. Topic focus
   1. Wrong agent
   2. Experimental data (*in vitro* or in *vivo* cellular, molecular, biochemical or other studies that do not include naturally occurring cases)
   3. Laboratory methods descriptions
   4. Campylobacteriosis included in the diagnostic evaluation or as a differential diagnosis, but diagnosis of *Campylobacter* infection was not reached.

**Full-text exclusion criteria (with coding hierarchy)**

1. Any texts where the country is not specified;
2. If two identical papers are published in different sources, the earliest paper/first report – based on the date of publication – should be selected for inclusion;
3. Methods or study population not described in sufficient detail to determine whether the study meets inclusion or exclusion criteria.

**Risk of bias (quality) assessment**

Risk of bias will be assessed separately for each eligible study, using an assessment tool [4]. This tool will be used to code and provide consistency for selection. We will decide to perform a meta-analysis (if data allows) according to the quality assessment scores and risk of bias assessments.

**Data extraction (selection and coding)**

Titles and abstracts will be screened for location, study population and general correlation with the research objectives. Full versions of potentially relevant articles will be obtained to assess eligibility. These will then be independently evaluated for inclusion. Cross-references of the full text retrieved articles will also be searched. Data will be collected independently from each publication and captured using a standardised Word document form. We plan to extract data from text, tables and figures. Study investigators will be contacted in cases of unclear data or eligibility criteria.

Data from eligible studies in humans will be extracted based on the following:

i. Methodological information; year of publication, country of study, study setting, year of research, study design (including target population), type of samples and sample size.

ii. Case definition: diagnostic methods used to prove *Campylobacter* spp.

iii. Study outcomes:

- Prevalence of human campylobacteriosis and 95% CI.
- *Campylobacter* species.

**Data synthesis**

Data will be collected independently from each publication and captured using a standardised form. Data will be extracted from text, tables and figures.

**Suggested data**

- Overview of epidemiological data on human campylobacteriosis in sub-Saharan Africa, sorted and described by region.

**Time schedule:**

| **Feb / Mar 2019** | Preparing protocol, reading literature & preparing search strategy.  Searching the literature. Screening of records by two independent reviewers |
| --- | --- |
| **Mar / Apr 2019** | Data extraction in standardized Word document form, data-entry, meta-analysis (if applicable) and drafting the manuscript. |
| **May - Aug 2019** | Finalizing manuscript and submit to peer-reviewed biomedical journal with preferably an open-access option. |
| **Aug - Oct 2019** | If applicable, revising the manuscript for re-submission. |

**Literature**

1. Wanger A, Chavez V, Huang RSP, Wahed A, Actor JK, Dasgupta A. Chapter 6 - Overview of Bacteria. Microbiology and Molecular Diagnosis in Pathology. 2017. P. 75-117.
2. Perez Perez GI, Kienesberger S. Chapter 9 - Campylobacter. Food Science and Technology 2013. P. 165-185.
3. Garza JM, Cohen MB. 39 - Infectious Diarrhea. Pediatric Gastrointestinal and Liver Disease (Fourth Edition) 2011. P. 405-422.e5
4. Hoy D, Brooks P, Woolf A, Blyth F, March L, Bain C, et al. Assessing Risk of Bias in Prevalence Studies: Modification of an Existing Tool and Evidence of Interrater Agreement.” Journal of Clinical Epidemiology. 2012; 65(9):934–39.

1. The UN macro-geographical definition of sub-Saharan Africa was used to define the geographic boundaries of this review <https://unstats.un.org/unsd/methodology/m49/> as the area covered by the following countries/territories:  Angola, Benin, Botswana, British Indian Ocean Territory, Burkina Faso, Burundi, Cabo Verde, Cameroon, Central African Republic, Chad, Comoros, Congo, Côte d’Ivoire, Democratic Republic of the Congo, Djibouti, Equatorial Guinea, Eritrea, Eswatini, Ethiopia, French Southern Territories, Gabon, Gambia, Ghana, Guinea, Guinea-Bissau, Kenya, Lesotho, Liberia, Madagascar, Malawi, Mali, Mauritania, Mauritius, Mayotte, Mozambique, Namibia, Niger, Nigeria, Réunion, Rwanda, Saint Helena, Sao Tome and Principe, Senegal, Seychelles, Sierra Leone, Somalia, South Africa, South Sudan, Togo, Uganda, United Republic of Tanzania, Zambia and Zimbabwe. [↑](#footnote-ref-1)
