# Supplementary material for: A systematic review and meta-analysis reveal that Campylobacter spp. and antibiotic resistance are widespread in humans in sub-Saharan Africa
Source: PLoS One. 2021 Jan 27;16(1):e0245951. doi: 10.1371/journal.pone.0245951 (PMC7840040; doi:10.1371/journal.pone.0245951)
Supplement: S2 File — (DOCX) [file pone.0245951.s002.docx]

**A systematic review and meta-analysis reveal that *Campylobacter* spp. and antibiotic resistance are widespread in humans in sub-Saharan Africa**

| **Searh Strategy** |
| --- |

**Update of 25 March 2020**

**Database: PubMed**

| **#** | **Research question** | **Records found** |
| --- | --- | --- |
| **#1** | Campylobacteriosis[Mesh] OR Campylobacter[Mesh] OR campylob*[tiab] | 18086 |
| **#2** | Africa South of the Sahara[Mesh] OR Africa south of the Sahara[tiab] OR sub-Saharan[tiab] OR subSaharan[tiab] OR Angola[tiab] OR Benin[tiab] OR Botswana[tiab] OR British Indian Ocean Territory[tiab] OR Burkina Faso[tiab] OR Burundi[tiab] OR Cape Verde[tiab] OR Cameroon[tiab] OR Central African Republic[tiab] OR Chad[tiab] OR Comoros[tiab] OR Congo[tiab] OR Cote d’Ivoire[tiab] OR Democratic Republic of the Congo[tiab] OR DRC[tiab] OR Zaire[tiab] OR Djibouti[tiab] OR Equatorial Guinea[tiab] OR Eritrea[tiab] OR Eswatini[tiab] OR Swaziland[tiab] OR Ethiopia[tiab] OR French Southern Territories[tiab] OR Gabon[tiab] OR Gambia[tiab] OR Ghana[tiab] OR (Guinea[tiab] NOT guinea pig*[tiab]) OR Guinea-Bissau[tiab] OR Kenya[tiab] OR Lesotho[tiab] OR Liberia[tiab] OR Madagascar[tiab] OR Malawi[tiab] OR Mali[tiab] OR Mauritania[tiab] OR Mauritius[tiab] OR Mayotte[tiab] OR Mozambique[tiab] OR Namibia[tiab] OR Niger[tiab] OR Nigeria[tiab] OR Reunion[tiab] OR Rwanda[tiab] OR Saint Helena[tiab] OR Sao Tome and Principe[tiab] OR Senegal[tiab] OR Seychelles[tiab] OR Sierra Leone[tiab] OR Somalia[tiab] OR South Africa[tiab] OR South Sudan[tiab] OR Togo[tiab] OR Uganda[tiab] OR Tanzania[tiab] OR United Republic of Tanzania[tiab] OR Zambia[tiab] OR Zimbabwe[tiab] OR Central Africa*[tiab] OR Eastern Africa*[tiab] OR East Africa*[tiab] OR Southern Africa*[tiab] OR Western Africa*[tiab] OR West Africa*[tiab] | 300992 |
| **#3** | #1 AND #2 | **393** |

**Database: Science Direct**

| ***#*** | ***Searches*** | ***Results*** |
| --- | --- | --- |
| **1** | Find articles with these terms: Campylobacter OR Campylobacteriosis Advanced search, Title, abstract, keywords: "Africa south of the Sahara" OR sub-Saharan OR subSaharan OR Angola OR Benin OR Botswana OR "British Indian Ocean Territory" OR "Burkina Faso" OR Burundi | 40 |
| **2** | Find articles with these terms: Campylobacter OR Campylobacteriosis Advanced search, Title, abstract, keywords: "Cape Verde" OR Cameroon OR "Central African Republic" OR Chad OR Comoros OR Congo OR "Cote d’Ivoire" OR "Democratic Republic of the Congo" OR DRC | 22 |
| **3** | Find articles with these terms: Campylobacter OR Campylobacteriosis Advanced search, Title, abstract, keywords: Zaire OR Djibouti OR "Equatorial Guinea" OR Eritrea OR Eswatini OR Swaziland OR Ethiopia OR "French Southern Territories" OR Gabon | 15 |
| **4** | Find articles with these terms: Campylobacter OR Campylobacteriosis Advanced search, Title, abstract, keywords: Gambia OR Ghana OR (Guinea NOT guinea pig) OR Guinea-Bissau OR Kenya OR Lesotho OR Liberia OR Madagascar | 54 |
| **5** | Find articles with these terms: Campylobacter OR Campylobacteriosis Advanced search, Title, abstract, keywords: Malawi OR Mali OR Mauritania OR Mauritius OR Mayotte OR Mozambique OR Namibia OR Niger OR Nigeria | 40 |
| **6** | Find articles with these terms: Campylobacter OR Campylobacteriosis Advanced search, Title, abstract, keywords: Reunion OR Rwanda OR "Saint Helena" OR "Sao Tome and Principe" OR Senegal OR Seychelles OR "Sierra Leone" OR Somalia OR "South Africa" | 71 |
| **7** | Find articles with these terms: Campylobacter OR Campylobacteriosis Advanced search, Title, abstract, keywords: "South Sudan" OR Togo OR Uganda OR Tanzania OR "United Republic of Tanzania" OR Zambia OR Zimbabwe OR "Central Africa" OR "Eastern Africa" | 39 |
| **8** | Find articles with these terms: Campylobacter OR Campylobacteriosis Advanced search, Title, abstract, keywords: "East Africa" OR "Southern Africa" OR "Western Africa" OR "West Africa" | 25 |
|  | Total | **306** |

**Article types: Research articles, Conference abstracts, Case reports, Short communications*

**Database: Google Scholar**

*(without patents and citations)*

| ***#*** | ***Searches*** | ***Results*** |
| --- | --- | --- |
| **1** | allintitle: campylobacteriosis \| campylobacter allintitle:"Africa south of the Sahara" \| sub-Saharan \| subSaharan \| Angola \| Benin \| Botswana \| "British Indian Ocean Territory" \| "Burkina Faso" \| Burundi \| "Cape Verde" \| Cameroon | 11 |
| **2** | allintitle: campylobacteriosis \| campylobacter allintitle:"Central African Republic" \| Chad \| Comoros \| Congo \| "Cote d’Ivoire" \| "Democratic Republic of the Congo" \| DRC \| Zaire \| Djibouti \| "Equatorial Guinea" \| Eritrea \| Eswatini \| Swaziland | 0 |
| **3** | allintitle: campylobacteriosis \| campylobacter allintitle:Ethiopia \| "French Southern Territories" \| Gabon \| Gambia \| Ghana \| Guinea \| Guinea-Bissau \| Kenya \| Lesotho \| Liberia \| Madagascar \| Malawi \| Mali \| Mauritania \| Mauritius \| Mayotte \| Mozambique | 42 |
| **4** | allintitle: campylobacteriosis \| campylobacter allintitle:Namibia \| Niger \| Nigeria \| Reunion \| Rwanda \| "Saint Helena" \| "Sao Tome and Principe" \| Senegal \| Seychelles \| "Sierra Leone" \| Somalia \| "South Africa" \| "South Sudan" \| Togo \| Uganda \| Tanzania | 164 |
| **5** | allintitle: campylobacteriosis \| campylobacter allintitle:"United Republic of Tanzania" \| Zambia \| Zimbabwe \| "Central Africa" \| "Eastern Africa" \| "East Africa" \| "Southern Africa" \| "Western Africa" \| "West Africa" | 9 |
|  | Total | **226** |

**Database: Cochrane Library**

| ***#*** | ***Searches*** | ***Results*** |
| --- | --- | --- |
| **#1** | Campylob* | 301 |
| **#2** | "Africa south of the Sahara" or sub-Saharan or subSaharan or Angola or Benin or Botswana or "British Indian Ocean Territory" or "Burkina Faso" or Burundi or "Cape Verde" or Cameroon or "Central African Republic" or Chad or Comoros or Congo or "Cote d’Ivoire" or "Democratic Republic of the Congo" or DRC or Zaire or Djibouti or "Equatorial Guinea" or Eritrea or Eswatini or Swaziland or Ethiopia or "French Southern Territories" or Gabon or Gambia or Ghana or (Guinea not guinea pig*) or Guinea-Bissau or Kenya or Lesotho or Liberia or Madagascar or Malawi or Mali or Mauritania or Mauritius or Mayotte or Mozambique or Namibia or Niger or Nigeria or Reunion or Rwanda or "Saint Helena" or "Sao Tome and Principe" or Senegal or Seychelles or "Sierra Leone" or Somalia or "South Africa" or "South Sudan" or Togo or Uganda or Tanzania or "United Republic of Tanzania" or Zambia or Zimbabwe or "Central Africa*" or "Eastern Africa*" or "East Africa*" or "Southern Africa*" or "Western Africa*" or "West Africa*" | 15542 |
| **#3** | #1 and #2 | **19** |

**Database: CINAHL**

| ***#*** | ***Searches*** | ***Results*** |
| --- | --- | --- |
| **1** | (MH "Campylobacteriosis") | 0 |
| **2** | TI (campylob*) | 710 |
| **3** | S1 OR S2 | 710 |
| **4** | TI ("Africa south of the Sahara" or sub-Saharan or subSaharan or Angola or Benin or Botswana or "British Indian Ocean Territory" or "Burkina Faso" or Burundi or "Cape Verde" or Cameroon or "Central African Republic" or Chad or Comoros or Congo or "Cote d’Ivoire" or "Democratic Republic of the Congo" or DRC or Zaire or Djibouti or "Equatorial Guinea" or Eritrea or Eswatini or Swaziland or Ethiopia or "French Southern Territories" or Gabon or Gambia or Ghana or (Guinea not guinea pig*) or Guinea-Bissau or Kenya or Lesotho or Liberia or Madagascar or Malawi or Mali or Mauritania or Mauritius or Mayotte or Mozambique or Namibia or Niger or Nigeria or Reunion or Rwanda or "Saint Helena" or "Sao Tome and Principe" or Senegal or Seychelles or "Sierra Leone" or Somalia or "South Africa" or "South Sudan" or Togo or Uganda or Tanzania or "United Republic of Tanzania" or Zambia or Zimbabwe or "Central Africa" or "Eastern Africa" or "East Africa" or "Southern Africa" or "Western Africa*" or "West Africa") OR AB ("Africa south of the Sahara" or sub-Saharan or subSaharan or Angola or Benin or Botswana or "British Indian Ocean Territory" or "Burkina Faso" or Burundi or "Cape Verde" or Cameroon or "Central African Republic" or Chad or Comoros or Congo or "Cote d’Ivoire" or "Democratic Republic of the Congo" or DRC or Zaire or Djibouti or "Equatorial Guinea" or Eritrea or Eswatini or Swaziland or Ethiopia or "French Southern Territories" or Gabon or Gambia or Ghana or (Guinea not guinea pig*) or Guinea-Bissau or Kenya or Lesotho or Liberia or Madagascar or Malawi or Mali or Mauritania or Mauritius or Mayotte or Mozambique or Namibia or Niger or Nigeria or Reunion or Rwanda or "Saint Helena" or "Sao Tome and Principe" or Senegal or Seychelles or "Sierra Leone" or Somalia or "South Africa" or "South Sudan" or Togo or Uganda or Tanzania or "United Republic of Tanzania" or Zambia or Zimbabwe or "Central Africa" or "Eastern Africa" or "East Africa" or "Southern Africa" or "Western Africa*" or "West Africa") | 64524 |
| **5** | S3 AND S4 | **10** |

**Database: African Index Medicus**

*(titles and keywords, all documents)*

| ***#*** | ***Searches*** | ***Results*** |
| --- | --- | --- |
| **1** | Campylobacteriosis | 2 |
| **2** | Campylobacter | 11 |
| **3** | 1 and 2 | **13** |

**Database: African Journals Online (AJOL)**

| ***#*** | ***Searches*** | ***Results*** |
| --- | --- | --- |
| **1** | campylob* | **38** |
