# Supplementary material for: A systematic review and meta-analysis reveal that Campylobacter spp. and antibiotic resistance are widespread in humans in sub-Saharan Africa
Source: PLoS One. 2021 Jan 27;16(1):e0245951. doi: 10.1371/journal.pone.0245951 (PMC7840040; doi:10.1371/journal.pone.0245951)
Supplement: S2 Table — (DOC) [file pone.0245951.s004.doc]

**A systematic review and meta-analysis reveal that *Campylobacter* spp. and antibiotic resistance are widespread in humans in sub-Saharan Africa**

**Risk of bias (quality) assessment for included human studies**. Adapted from Hoy *et al*., 2012.

| **Refe**  **re**  **nce** | **External validity** | | | | **Internal validity** | | | | | | **Summary overall risk of study bias (10 lowest risk)** |
| --- | --- | --- | --- | --- | --- | --- | --- | --- | --- | --- | --- |
|  | **Target population -close representation of the regional population?** | **Sampling frame true/close representation of population**  **AND / OR**  **in- and exclusion criteria defined?** | **Adequate selection of participants** | **Likelihood of nonresponse bias minimal? (not applicable to cross sectional studies, only cohort)** | **Were data collected directly from subjects?** | **Acceptable case definition used?** | **Study instrument valid & reliable?** | **Same mode of data collection from all subjects?** | **Was the length of the shortest prevalence**  **period for the**  **parameter of interest appropriate?** | **Numerator/denominator appropriate?** |  |
| **Eastern Africa** | | | | | | | | | | | |
| [1] | No  One hospital | Yes  Clear inclusion criteria | Yes  All attending inclusion criteria | Not applicable  Cross-sectional study | Yes | Yes | Yes  Culture, microscopy and biochemical tests | Yes | Yes  September 2002 - June 2003 | Yes | **8** |
| [2] | No  Selection of villages by convenience | Yes  Clear inclusion criteria | Yes  All attending inclusion criteria | Not applicable  Cross-sectional study | Yes | Yes | Yes  Culture and microscopy | Yes | Yes  April - May 1998 | Yes | **8** |
| [3] | Yes  One hospital and one health centre | Yes  Clear inclusion criteria | Yes  All attending inclusion criteria | Not applicable  Cross-sectional study | Yes | Yes | Yes  Culture, microscopy and biochemical tests | Yes | Yes  July - October 2012 | Yes | **9** |
| [4] | No  One hospital | Yes  Clear inclusion criteria | Yes  All attending inclusion criteria | Not applicable  Cross-sectional study | Yes | Yes | Yes  Culture, microscopy and biochemical tests | Yes | Yes  October 2007 - April 2008 | Not clear  (no numerator and discordant data) | **7** |
| [5] | Yes  One hospital and one health centre | Yes  Clear inclusion and exclusion criteria | Yes  All attending inclusion criteria | Not applicable  Cross-sectional study | Yes | Yes | Yes  Culture, microscopy and biochemical tests | No  Stool samples and rectal swabs | Yes  June 6 - October 28, 2011 | Yes | **8** |
| [6] | Yes  Study area was a hospital | Yes  Clear inclusion and exclusion criteria | No  Convenience sampling | Not applicable  Cross-sectional study | Yes | Yes | Yes  Culture, microscopy and biochemical tests | Yes | Yes  October 2011 -March 2012 | Yes | **8** |
| [7] | Yes  Study area was hospital | Yes  Clear inclusion and exclusion criteria | Yes  All attending inclusion criteria | Not applicable  Cross-sectional study | Yes | Yes | Yes  Culture and biochemical tests | Yes | Yes  February - May, 2016 | Yes | **9** |
| [8] | Yes 3 sites in Asembo | Yes  Clear inclusion criteria | Yes  All attending inclusion criteria | Not applicable  Cross-sectional study | Yes | Not clear diagnostic method (culture medium) | Not clear | No  Stool samples and rectal swabs | Yes  May 1997 - April 1998 | Yes | **6** |
| [9] | No  One Medical Clinic | Yes  Clear inclusion criteria | Yes  All attending inclusion criteria | Not applicable  Cross-sectional study | Yes | Not clear diagnostic method (culture medium) | Not clear | Yes | Yes  1^st^ November 2008 -31^st^ December 2011 | Not clear (only percentage, no numerator) | **5** |
| [10] | No  One outpatient clinic of a general hospital | Yes  Clear inclusion and exclusion criteria | Yes | Yes  Response rate for the study was ≥75% | Yes | Not clear diagnostic method (culture medium) | Not clear | No  Stool samples and rectal swabs | Yes  1997 - 2001 | Yes | **6** |
| [11] | Yes  An area of the district | Yes  Clear inclusion and exclusion criteria | Yes  All attending inclusion criteria | Not applicable  Case-control study | Yes | Not clear diagnostic method (culture medium) | Not clear | Yes | Yes  May 1997 - September 2001 | Yes | **7** |
| [12] | No  Two hospitals | Yes  Clear inclusion and exclusion criteria | Yes  All attending inclusion criteria | Not applicable  Cross-sectional study | Yes | Yes | Yes  Culture | No  Stool samples and rectal swabs | Yes  November 2011 – October 2013 | Yes | **7** |
| [13] | Yes  Two hospitals in Nyanza province | Yes  Clear inclusion and exclusion criteria | Yes  All attending inclusion criteria | Not applicable  Cross-sectional study | Yes | Not clear diagnostic method (culture medium) | Not clear | No  Stool samples and rectal swabs | Yes  November 2011 - June 2014 | Not clear | **5** |
| [14] | Yes  Study area was a hospital | Yes  Clear inclusion criteria | Not clear | Not applicable  Cross-sectional study | Yes | Not clear diagnostic method | Not clear | Yes | Not clear | Not clear (no numerator) | **4** |
| [15] | Yes  Two large government- operated district hospitals | Yes  Clear inclusion criteria | Yes  All attending inclusion criteria | Yes  Response rate for the study was ≥75%. 10% died. | Yes | Yes | Yes  Culture and biochemical tests | No  Stool samples and rectal swabs | Yes  May 23, 2005 - May 22, 2007 | Yes | **9** |
| [16] | No  One outpatient clinic of a hospital (urban area) and four outpatient clinics (rural area) | Yes  Clear inclusion criteria | No  Different sampling methods in rural and urban area | Not applicable  Cross-sectional study | Yes | Not clear diagnostic method (culture medium) | Not clear | Yes | Yes  1997 - 2003 | Yes | **5** |
| [17] | Yes  Two rural clinics; one outpatient clinic of a hospital and one private clinic | Yes  Clear inclusion criteria | Yes  All attending inclusion criteria | Not applicable  Cross-sectional study | Yes | Not clear diagnostic method (culture medium | Not clear | No  Stool samples and rectal swabs | Yes  May 1997 - April 2003 | No  Prevalence should be 8.5%, not 8% | **5** |
| [18] | Yes  Two district hospitals | Yes  Clear inclusion criteria | Not clear | Not applicable  Case-control study | Yes | Yes | Yes  Culture | Yes | Yes  September 2009 - September 2011 | Yes | **8** |
| [19] | No  One hospital | Yes  Clear inclusion criteria | Yes  All attending inclusion criteria | Not applicable  Case-control study | Yes | Yes | Yes  Culture, microscopy and biochemical tests | Yes | Yes  November 4, 2009 - February 4, 2011 | Yes | **8** |
| [20] | Yes,  Eleven schools | Yes  Clear inclusion criteria | Yes  Random selection | Not applicable  Cross-sectional study | Yes | Yes | Yes  Culture, biochemical tests and PCR | Yes | Yes  July, 2015 | Yes | **9** |
| [21] | No  Two villages | Yes  Clear inclusion criteria | Yes  All attending inclusion criteria | Yes  Response rate for the study was ≥75%. 13.4% drop out | Yes | Yes | Yes  Culture, haemagglutination test and PCR | Yes | Yes  January 2010 – May 31^st^ 2012 | Yes | **9** |
| [22] | Yes  Two hospitals | Yes  Clear inclusion criteria | Yes  All attending inclusion criteria | Not applicable  Matched Case-control study | Yes | Yes | Yes  Culture and haemagglutination test | Yes | Yes  November 2011 - January 2014 | Yes | **9** |
| [23] | Yes  14 locations that are representative  (Communities) | Yes  Clear inclusion and exclusion criteria | Yes  All attending inclusion criteria | Not applicable  Case-control study | Yes | Yes | Yes  Culture and haemaglutination test | Yes | Yes  February 2008 – May 2009 | Yes | **9** |
| [24] | No  One hospital | Yes  Clear inclusion criteria | Yes  All attending inclusion criteria | Not applicable  Case-control study | Yes | Yes | Yes  PCR | Yes | Yes  1997 – 2007 | Yes | **8** |
| [25] | No  One hospital | Yes  Clear inclusion and exclusion criteria | Yes  From another study | Yes  Response rate for the study was ≥75% | Yes | Yes | Yes  PCR | Yes | Yes  January - July 2013 | Yes | **9** |
| [26] | No  One hospital | Yes  Clear inclusion and exclusion criteria | Yes  From another study | Yes  Response rate for the  study was ≥75%.  Mortality rate of 17% | Yes | Yes | Yes  PCR | Yes | Yes  January - July 2013 | Yes | **9** |
| [27] | Yes  District hospital | Yes  Clear inclusion criteria | Yes  One out of every two hospitalized children | Not applicable  Cross-sectional study | Yes | Yes | Yes  Culture, microscopy and biochemical tests | Yes | Yes  September 2000 - September 2001 | Yes | **9** |
| [28] | Yes  Low-income, unplanned neighbourhoods | Yes  Clear inclusion criteria | Yes  All attending inclusion criteria | Not applicable  Cross-sectional study | Yes | Yes | Yes  PCR | Yes | Yes  February 2015 - February 2016 | Numerator not available | **8** |
| [29] | Yes  Health facilities at Manhiça District | Yes  Clear inclusion criteria | Yes  All attending inclusion criteria | Not applicable  Cross-sectional study | Yes | Yes | Yes  Culture, microscopy and biochemical tests | Yes | Yes  December 2007 - October 2011 | Not clear for prevalence in controls | **8** |
| [30] | Yes  Three health centres | Yes  Clear inclusion and exclusion criteria | Yes  All attending inclusion criteria | Not applicable  Cross-sectional study | Yes | Yes | Yes  PCR | No  Stool samples and rectal swabs | Yes  November 2009 - June 2012 | Yes | **8** |
| [31] | Yes  Two health centres, two district hospitals and two university hospitals | Yes  Clear inclusion criteria | Yes  All attending inclusion criteria | Not applicable  Case-control study | Yes | Yes | Yes  PCR | Yes | Yes  2011–2012 | Yes | **9** |
| [32] | No  One hospital | Yes  Clear inclusion and exclusion criteria | Yes  All attending inclusion criteria | Not applicable  Cross-sectional study | Yes | Yes | Yes  Culture, microscopy and biochemical tests | Yes | Yes  July - October 2005 | Yes | **8** |
| [33] | Yes  Two hospitals and four health centres | Yes  Clear inclusion and exclusion criteria | Yes  All attending inclusion criteria | Not applicable  Cross-sectional study | Yes | Yes | Yes  Culture, microscopy, biochemical tests and PCR | Yes | Yes  December 2006 - May 2007 | Yes | **9** |
| [34] | No  One clinic | Yes  Clear inclusion and exclusion criteria | No  Convenience sampling | Not applicable  Cross-sectional study | Yes | Yes | Yes  PCR | No  Fecal swab, stool collected from diaper or collection container (pot) | Yes  January 2009 -January 2011 | Yes | **6** |
| [35] | Yes  Regional hospital | Yes  Clear inclusion and exclusion criteria | Yes  All attending inclusion criteria | Not applicable  Case-control study | Yes | Yes | Yes  Culture, microscopy and biochemical tests | Yes | Yes  January - September 2011 | Yes | **9** |
| [36] | Yes  Two hospitals: referral and regional | Yes  Clear inclusion criteria | Yes  All attending inclusion criteria | Not applicable  Cross-sectional study | Yes | Yes | Yes  Culture and microscopy | Yes | Yes  October 2012 - April 2013 | Yes | **9** |
| [37] | Yes  Six villages, three health centres | No | Not clear | Not applicable  Cross-sectional study | Yes | Yes | Yes  Culture, microscopy and biochemical tests | Yes | Not clear | Yes | **6** |
| [38] | No  Villages selected conveniently; one hospital, one university, one laboratory | Yes  Clear inclusion criteria | Yes  All attending inclusion criteria | Not applicable  Cross-sectional study | Yes | Yes | Yes  Culture, microscopy, biochemical tests and PCR | Yes | Yes  January 2003 - December 2004. | Yes | **8** |
| [39] | Yes  One hospital, one dispensary and one medical laboratory | Yes  Clear inclusion criteria | Yes  All attending inclusion criteria | Not applicable  Cross-sectional study | Yes | Yes | Yes  Culture, microscopy, biochemical tests and PCR | Yes | Yes  December 2011 - April 2012 | Yes | **9** |
| [40] | No  One Primary Health Care Centre | Yes  Clear inclusion and exclusion criteria | Yes  All attending inclusion criteria | Not applicable  Case-control study | Yes | Yes | Yes  PCR | Yes | Yes  April - July 2011 | Yes | **8** |
| [41] | No  One primary health centre | Yes  Clear inclusion criteria | Yes  All attending inclusion criteria | Yes  100% on follow up (Cohort study) | Yes | Yes | Yes  PCR | Yes | Yes  April – July 2011 | Yes | **9** |
| [42] | Yes  Study are was a hospital | Yes  Clear inclusion criteria | Yes  All attending inclusion criteria | Not applicable  Cross-sectional study | Yes | Yes | Yes  Culture and biochemical tests | Yes | Yes  December 2015 - April 2016 | Yes | **9** |
| [43] | Yes  Four hospitals | Yes  Clear inclusion and exclusion criteria | Yes  All attending inclusion criteria | Not applicable  Cross-sectional study | Yes | Yes | Yes  Culture | Yes | Yes  December 1999 - March 2000 | Yes | **9** |
| **Middle Africa** | | | | | | | | | | | |
| [44] | No  One hospital | Yes  Clear inclusion criteria | Yes  All attending inclusion criteria | Not applicable  Case-control study | Yes | Yes | Yes  PCR | Yes | Yes  December 25, 2013- October 5, 2014 | Yes | **8** |
| **Southern Africa** | | | | | | | | | | | |
| [45] | No  Four sites in Botswana (hospitals in one city, one town and two villages). | Yes  Clear inclusion criteria | No  Convenience sampling | Not applicable  Cross-sectional study | Yes | Yes | Yes  Culture and microscopy | Yes | Yes  March 2001 - October 2003 | Yes | **7** |
| [46] | Yes  Princess Marina Hospital, clinics and smaller hospitals | Yes  Clear inclusion and exclusion criteria | Yes  All attending inclusion criteria | Not applicable  Retrospective Cross-sectional study | Yes | Not clear diagnostic method (medium) | Not clear | Yes | Yes  February 2003 - July 2008 | Yes | **7** |
| [47] | Yes  Two hospitals | Yes  Clear inclusion and exclusion criteria | Yes  All attending inclusion criteria | Yes  Response rate for the study was ≥75%  26 deaths (3.9%) | Yes | Yes | Yes  PCR | Yes | Yes  May 2011 - April 2013 | Yes | **10** |
| [48] | No  One hospital | Yes  Clear inclusion and exclusion criteria | Yes  All attending inclusion criteria | Not applicable  Cross-sectional study | Yes | Yes | Yes  Culture | Yes | Yes  2000 - 2002 | Yes  (Some species are currently not included in  *Campylobacter* genus) | **8** |
| [49] | Yes  Followed the WHO guidelines for surveillance | Yes  Clear inclusion and exclusion criteria | Yes  All attending inclusion criteria | Not applicable  Cross-sectional study | Yes | Yes | Yes  PCR | Yes | Yes  October 2014 - December 2015 | Yes | **9** |
| [50] | No | Yes  Clear inclusion criteria | Not clear | Not applicable  Cross-sectional study | Yes | Yes | Yes  Culture and biochemical tests | Yes | Yes  March 2001 –February 2002 | Yes | **7** |
| [51] | Yes  Three hospitals and two schools | Yes  Clear inclusion criteria | Yes  All attending inclusion criteria | Not applicable  Cross-sectional study | Yes | Yes | Yes  Culture, haemmaglutination tests and PCR | Yes | Yes  November 2004 - May 2005 | Yes | **9** |
| [52] | Not clear – sub study | Yes  Clear inclusion and exclusion criteria | Yes  All attending inclusion criteria | Not applicable  Cross-sectional study | Yes | Yes | Yes  PCR | Yes | Yes  March 2012 - March 2013 | Yes | **8** |
| [53] | Yes  Two primary schools and three main public hospitals | Yes  Clear inclusion criteria | Yes  Random sampling | Not applicable  Cross-sectional study | Yes | Not clear diagnostic method (culture medium) | Not clear | Yes | Yes  October 2003 - April 2005 | Yes | **7** |
| [54] | Yes  Four major public hospitals in the district | Yes  Clear inclusion criteria | Yes  All attending inclusion criteria | Not applicable  Cross-sectional study | Yes | Yes | Yes  Culture, microscopy, biochemical tests, haemmaglutination tests, PCR | Yes | Yes  November 2004 - May 2005 | No  Prevalence should be 20.4% not 20.3% | **8** |
| [55] | Yes  Local Community Health Centre | Yes  Clear inclusion criteria | Yes  All attending inclusion criteria | Not applicable  Cross-sectional study | Yes | Yes | Yes  Culture | Yes | Yes  October 2015 – April 2016 | Yes | **9** |
| **Western Africa** | | | | | | | | | | | |
| [56] | No  One of the four secondary health care centres in Ouagadougou | Yes  Clear inclusion criteria | Yes  All attending inclusion criteria | Not applicable  Case-control study | Yes | Yes | Yes  Culture | Yes | Yes  January 2009 - January 2010 | Yes | **8** |
| [57] | No  One hospital | Yes  Clear inclusion criteria | Yes  All attending inclusion criteria | Not applicable  Cross-sectional study | Yes | Yes  Stool | Yes  Culture, microscopy and biochemical tests | Yes | Yes  November 2006 - February 2008 | Yes | **8** |
| [58] | No  One clinic and one medical centre | Not clear | Not clear | Not applicable  Cross-sectional study | Yes | Yes | Yes  PCR | Yes | Yes  February 5th - March 9th, 2013 | Yes | **6** |
| [59] | Yes  One hospital and village healthcare centres | Yes  Clear inclusion and exclusion criteria | Yes  All attending inclusion criteria | Not applicable  Case-control study | Yes | Yes | Yes  PCR | Yes | Yes  October 2012 | Yes | **9** |
| [60] | Yes  Second-largest hospital in Ghana and the only tertiary health institution in the region | Yes  Clear inclusion  criteria | Yes  All attending inclusion criteria | Not applicable  Cross-sectional study | Yes | Yes | Yes  Culture, microscopy and biochemical tests | No  Stool from some subjects and urine for others | Yes  May - August 2013 | Yes | **8** |
| [61] | No  One hospital | Yes  Clear inclusion and exclusion criteria | Yes  All attending inclusion criteria | Not applicable  Case-control study | Yes | Yes | Yes  PCR | Yes | Yes  June 2007 - October 2008 | Yes | **8** |
| [62] | Yes  Two neighbouring areas | Yes  Clear inclusion criteria | Yes  All attending inclusion criteria | Not applicable  Cross-sectional study | Yes | Not clear diagnostic method (culture medium) | Not clear | Yes | Yes  January - April 2001 | Yes | **7** |
| [63] | No  One hospital and 10 health centres in one region | Yes  Clear inclusion criteria | Yes  All attending inclusion criteria | Not applicable  Cross-sectional study | Yes | Yes | Yes  Culture, microscopy and biochemical tests | Yes | Yes  April 2010 - March 2012 | Yes | **8** |
| [64] | Yes  Hospitals and health centres | Yes  Clear inclusion criteria | YeYes  All attending inclusion criteria | Not applicable  Case-control study | Yes | Yes | Yes  Culture, microscopy and biochemical tests | No  Stool samples and rectal swabs | Yes  December 2002 - November 2003 | Yes | **8** |
| [65] | No  Randomly selected hospitals | No | Not clear | Not applicable  Cross-sectional study | Yes | Yes | Yes  Culture, microscopy and biochemical tests | Not clear | Not reported | Yes | **4** |
| [66] | No  One hospital | Yes  Clear inclusion criteria | Yes  All attending inclusion criteria | Not applicable  Cross-sectional study | Yes | Yes | Yes  Culture | Yes | Yes  May 2011 - April, 2014 | Yes | **8** |
| [67] | No  One local hospital | Yes  Clear inclusion criteria | Yes  All attending inclusion criteria | Not clear (Prospective study) | Yes | Not clear diagnostic method (culture medium) | Not clear | Yes | Not reported | No %, only numerator and denominator | **4** |
| [68] | Not clear | Yes  Clear inclusion criteria | Not clear | Not applicable  Case-control study | Yes | Not clear | Not clear | No  Stool samples and rectal swabs | Not clear | Yes | **3** |
| [69] | No  Pig rearing areas | Yes  Clear inclusion criteria | No  Convenience sampling | Not applicable  Cross-sectional study | Yes | Yes | Yes  Culture, microscopy and biochemical tests | Yes | Yes  September, 2013 - February, 2014 | Yes | **7** |
| [70] | Yes  One hospital and one health centre | Yes  Clear inclusion criteria | Yes  Random selection | Not applicable  Case-control study | Yes | Yes | Yes  Culture, microscopy and biochemical tests | No  Stool samples and rectal swabs | Yes  April - December 1997 | Yes | **8** |
| [71] | No  One hospital | Yes  Clear inclusion criteria | Not clear | Not applicable  Cross-sectional study | Yes | Yes | Yes  Culture | Yes | No  2002 - 2006 | Yes | **6** |
| [72] | Yes  One hospital and one health centre | Yes  Clear inclusion and exclusion criteria | Yes  All attending inclusion criteria | Not applicable  Case-control study | Yes | Yes | Yes  Culture, microscopy and biochemical tests | No  Stool samples and rectal swabs | Not clear | Yes | **7** |
| [73] | No  One hospital | Yes  Clear inclusion criteria | Yes  All attending inclusion criteria | Not applicable  Case-control study | Yes | Yes | Yes  Culture, microscopy and biochemical tests | No  Stool samples and rectal swabs | Yes  September 2012 - March 2013 | Yes | **7** |
| [74] | No  One hospital and an Institute | Yes  Clear inclusion criteria | Not clear | Not applicable  Cross-sectional study | Yes | Yes | Yes  Culture, microscopy and biochemical tests | Yes | Yes  February - March  2017 | Yes | **7** |
| [75] | Yes  Five hospitals | Yes  Clear inclusion criteria | Yes  All attending inclusion criteria | Not applicable  Cross-sectional study | Yes | Yes | Yes  Culture, biochemical tests and PCR | Yes | Yes  August - December 2017 | Yes | **9** |
| [76] | Yes  Primary health care organisation for maternal/child health in Rebeuss, a poor and densely populated district of central Dakar | Yes  Clear inclusion criteria | Yes  All attending inclusion criteria | Not applicable  Cross-sectional study | Yes | Yes | Yes  Culture | Yes | Yes  September 2007 - March 2008 | Yes | **9** |
| [77] | Yes  Two hospitals | Yes  Clear inclusion criteria | Yes  All attending inclusion criteria | Not applicable  Case-control study | Yes | Not clear diagnostic method (culture medium) | Not clear | Yes | Not clear | Yes | **6** |

**CODING MANUAL FOR EPIDEMIOLOGICAL STUDIES**

(*Adapted from Hoy and colleagues* [78])

*External validity*

**1. Was the target population a close representation of the regional population / was it a population-based study?**

The target population refers to the group of people or entities to which the results of the study will be generalised. Examples:

- The study was a national health survey of people and the sample was drawn from a list that included all individuals in the population. The answer is: Yes (LOW RISK).
- The study was conducted in one or two main hospitals, serving a complete region with a representative population for the region. The answer is: Yes (LOW RISK).
- The study was undertaken in one village only and it is clear this was not representative of the national population. The answer is: No (HIGH RISK).

**2. Sampling frame true/close representation of the target population and / or in- and exclusion criteria defined.**

The sampling frame is a list of the sampling units in the target population and the study sample is drawn from this list. Examples:

- The sampling frame was a list of almost every individual within the target population and/or clear inclusion criteria. The answer is: Yes (LOW RISK).
- A probabilistic sampling method was used. The answer is: Yes (LOW RISK).
- The sampling frame is not clear and/or not clear in-and exclusion criteria. The answer is: No (HIGH RISK).

**3. Adequate selection of participants**

A census collects information from every unit in the sampling frame. In a survey, only part of the sampling frame is sampled. In these instances, random selection of the sample helps minimize study bias. Examples:

- The sample was selected using simple random sampling. The answer is: Yes (LOW RISK).
- The target population was all participants attending inclusion criteria. The answer is: Yes (LOW RISK).
- A convenience selection of participants was applied. The answer is: No (HIGH RISK).

**4. Likelihood nonresponse bias minimal? (not applicable to cross sectional studies, only cohort)**

There is a low risk when either the response rate for the study was ≥75%, OR, an analysis was performed that showed no significant difference in relevant demographic characteristics between responders and non-responders.

*Internal validity*

**5. Were data collected directly from the subjects?**

To prevent bias, all data should be collected directly from the subjects.

**6. Was an acceptable case definition used in the study?**

Laboratory-confirmed cases of *Campylobacter* spp. were defined as:

Clinical signs and symptoms consistent with campylobacteriosis and any one of the following:

- Detection of *Campylobacter* spp. in clinical samples by culture, microscopy technique and/or biochemical tests;
- *Campylobacter* spp. DNA detected by PCR.

**7. Was the study instrument that measured the parameter of interest shown to have validity and reliability?**

The following study instruments are considered valid & reliable:

- PCR;
- Culture, microscopy and biochemical tests.

**8. Was the same mode of data collection used for all subjects?**

To prevent bias, all data should be collected in the same way.

**9. Was the length of the shortest prevalence period for the parameter of interest appropriate?**

Investigations of less than one year are more likely to introduce higher estimates of disease because they were most likely performed during epidemics or in periods of high seasonal transmission.

__Yes (LOW RISK): The shortest prevalence period for the parameter of interest was appropriate (e.g., one-week prevalence, one-year prevalence).

__No (HIGH RISK): The shortest prevalence period for the parameter of interest was not appropriate (e.g., lifetime prevalence).

**10. Were the numerator(s) and denominator(s) for the parameter of interest appropriate?**

There may be errors in the calculation and/or reporting of the numerator and/or denominator.

**Reference list**

1. Beyene G, Haile-Amlak A. Antimicrobial sensitivity pattern of *Campylobacter* species among children in Jimma University Specialized Hospital, Southwest Ethiopia. Ethiop J Heal Dev. 2004;18: 185–189. doi:10.4314/ejhd.v18i3.9958

2. Mitike G, Kassu A, Genetu A, Nigussie D. *Campylobacter* enteritis among children in Dembia District, Northwest Ethiopia. East Afr Med J. 2000;77: 654–657. doi:10.4314/eamj.v77i12.46764

3. Tafa B, Sewunet T, Tassew H, Asrat D. Isolation and Antimicrobial Susceptibility Patterns of *Campylobacter* Species among Diarrheic Children at Jimma, Ethiopia. Int J Bacteriol. 2014;2014: 1–7. doi:10.1155/2014/560617

4. Ewnetu D, Muhret A. Prevalence and Antimicrobial Resistance of *Campylobacter* Isolates from Humans and Chickens in Bahir Dar, Ethiopia. Foodborne Pathog Dis. 2010;7: 667–670. doi:10.1089/fpd.2009.0433

5. Mulatu G, Getenet B, Ahmed Z. Prevalence of *Shigella, Salmonella* and *Campylobacter* species and their susceptibility patters among under five children with diarrhea in Hawassa Town, South Ethiopia. Ethiop J Heal Sci. 2014;24: 101–108. doi:10.4314/ejhs.v24i2.1

6. Lengerh A, Moges F, Unakal C, Anagaw B. Prevalence, associated risk factors and antimicrobial susceptibility pattern of *Campylobacter* species among under five diarrheic children at Gondar University Hospital, Northwest Ethiopia. BMC Pediatr. 2013;13. doi:10.1186/1471-2431-13-82

7. Kebede A, Aragie S, Shimelis T. The common enteric bacterial pathogens and their antimicrobial susceptibility pattern among HIV-infected individuals attending the antiretroviral therapy clinic of Hawassa university hospital, southern Ethiopia. Antimicrob Resist Infect Control. 2017;6: 1–7. doi:10.1186/s13756-017-0288-7

8. Shapiro RL, Kumar L, Phillips-Howard P, Wells JG, Adcock P, Brooks J, et al. Antimicrobial‐Resistant Bacterial Diarrhea in Rural Western Kenya. J Infect Dis. 2001;183: 1701–1704. doi:10.1086/320710

9. Mogeni DO, Otieno CL, Awiti G, Wamola N, Fields B, Neatherlin J, et al. Detection of viral respiratory and gastrointestinal pathogens among healthy adults and children of an informal settlement (Kibera) in Nairobi, Kenya. Int J Infect Dis. 2014;21: 223. doi:10.1016/j.ijid.2014.03.885

10. van Eijk A., Brooks JT, Adcock PM, Garrett V, Eberhard M, Rosen DH, et al. Diarrhea in children less than two years of age with known HIV status in Kisumu, Kenya. Int J Infect Dis. 2010;14: e220–e225. doi:10.1016/j.ijid.2009.06.001

11. Brooks JT, Shapiro RL, Kumar L, Wells JO, Phillips-Howard PA, Shi Y-P, et al. Epidemiology of sporadic bloody diarrhea in Rural Western Kenya. Am J Trop Med Hyg. 2003;68: 671–677.

12. Pavlinac PB, John-Stewart GC, Naulikha JM, Onchiri FM, Denno DM, Odundo EA, et al. High-Risk Enteric Pathogens Associated with HIV-Infection and HIV-Exposure in Kenyan Children with Acute Diarrhea. AIDS. 2014;28: 2287–2296. doi:10.1097/QAD.0000000000000396

13. Tickell KD, Pavlinac PB, John-Stewart GC, Denno DM, Richardson BA, Naulikha JM, et al. Impact of Childhood Nutritional Status on Pathogen Prevalence and Severity of Acute Diarrhea. Am J Trop Med Hyg. 2017;97: 1337–1344. doi:10.4269/ajtmh.17-0139

14. Ongwae ZH, Mwamburi LA, Kakai R. Multiple drug resistance of *Campylobacter jejuni* and *Shigella* isolated from diarrhoeic children aged under five years admitted at Kapsabet County Hospital, Kenya. Proceedings of the 2018 International Women in Science Without Borders (WISWB) – Indaba. Johannesburg, South Africa; 2018. pp. 1–3.

15. O’Reilly CE, Jaron P, Ochieng B, Nyaguara A, Tate JE, Parsons MB, et al. Risk Factors for Death among Children Less than 5 Years Old Hospitalized with Diarrhea in Rural Western Kenya , 2005–2007: A Cohort Study. PLoS Med. 2012;9: 2005–2007. doi:10.1371/journal.pmed.1001256

16. Beatty ME, Ochieng JB, Chege W, Kumar L, Okoth G, Shapiro RL, et al. Sporadic paediatric diarrhoeal illness in urban and rural sites in Nyanza province, Kenya. East Afr Med J. 2009;86: 387–398.

17. Brooks JT, Ochieng JB, Kumar L, Okoth G, Shapiro RL, Wells JG, et al. Surveillance for Bacterial Diarrhea and Antimicrobial Resistance in Rural Western Kenya, 1997–2003. Clin Infect Dis. 2006;43: 383–401. doi:10.1086/505866

18. Swierczewski BE, Odundo EA, Koech MC, Ndonye JN, Kirera RK, Odhiambo CP, et al. Surveillance for enteric pathogens in a case-control study of acute diarrhea in Western Kenya. Trans R Soc Trop Med Hyg. 2013;107: 83–90. doi:10.1093/trstmh/trs022

19. Conan A, O’Reilly CE, Ogola E, Ochieng JB, Blackstock AJ, Omore R, et al. Animal-related factors associated with moderate-to-severe diarrhea in children younger than five years in western Kenya: A matched case-control study. PLoS Negl Trop Dis. 2017;11: e0005795. doi:10.1371/journal.pntd.0005795

20. Gitahi N, Gathura PB, Gicheru MM, Wandia BM, Nordin A. Multidrug-resistant *Campylobacter jejuni,* *Campylobacter coli* and *Campylobacter lari* isolated from asymptomatic school-going children in Kibera slum, Kenya [version 2; peer review: 1 approved, 1 approved with reservations]. F1000Research. 2020;9. doi:10.12688/f1000research.21299.2

21. Randremanana RV, Randrianirina F, Sabatier P, Rakotonirina HC, Randriamanantena A, Razanajatovo IM, et al. *Campylobacter* infection in a cohort of rural children in Moramanga, Madagascar. BMC Infect Dis. 2014;14. doi:10.1186/1471-2334-14-372

22. Randremanana RV, Razafindratsimandresy R, Andriatahina T, Randriamanantena A, Ravelomanana L, Randrianirina F, et al. Etiologies, Risk Factors and Impact of Severe Diarrhea in the Under-Fives in Moramanga. PLoS One. 2016;11: e0158862. doi:10.1371/journal.pone.0158862

23. Randremanana RV. Impacts de l’environnement sur les diarrhées infantiles à Madagascar: Analyse du risque *Campylobacter*. Université de Grenoble. 2013. Available: https://tel.archives-ouvertes.fr/tel-00872059/document

24. Mason J, Iturriza-Gomara M, O’Brien SJ, Ngwira BM, Dove W, Maiden MCJ, et al. *Campylobacter* Infection in Children in Malawi Is Common and Is Frequently Associated with Enteric Virus Co-Infections. PLoS One. 2013;8: e59663. doi:10.1371/journal.pone.0059663

25. Versloot CJ, Attia S, Bourdon C, Richardson SE, Potani I, Bandsma RHJ, et al. Intestinal pathogen clearance in children with severe acute malnutrition is unrelated to inpatient morbidity. Clin Nutr ESPEN. 2018;24: 109–113. doi:10.1016/j.clnesp.2018.01.004

26. Attia S, Versloot CJ, Voskuijl W, Vliet SJV, Giovanni VD, Zhang L, et al. Mortality in children with complicated severe acute malnutrition is related to intestinal and systemic inflammation: an observational cohort study. Am J Clin Nutr. 2016;104: 1441–1449. doi:10.3945/ajcn.116.130518

27. Mandomando IM, Macete EV, Ruiz J, Sanz S, Abacassamo F, Vallès X, et al. Etiology of diarrhea in children younger than 5 years of age admitted in a rural hospital of Southern Mozambique. Am J Trop Med Hyg. 2007;76: 522–527.

28. Knee J, Sumner T, Adriano Z, Berendes D, Bruijn E, Schmidt W-P, et al. Risk factors for childhood enteric infection in urban Maputo, Mozambique: A cross-sectional study. PLoS Negl Trop Dis. 2018;12: e0006956. doi:10.1371/journal.pntd.0006956

29. Nhampossa T, Mandomando I, Acacio S, Quintó L, Vubil D, Ruiz J, et al. Diarrheal Disease in Rural Mozambique: Burden, Risk Factors and Etiology of Diarrheal Disease among Children aged 0–59 Months Seeking Care at Health Facilities. PLoS One. 2015;10: e0119824. doi:10.1371/journal.pone.0119824

30. Kabayiza J-C, Andersson ME, Nilsson S, Baribwira C, Muhirwa G, Bergström T, et al. Diarrhoeagenic microbes by real-time PCR in Rwandan children under 5 years of age with acute gastroenteritis. Clin Microbiol Infect. 2014;20: O1128–O1135. doi:10.1111/1469-0691.12698

31. Kabayiza J-C, Andersson ME, Nilsson S, Bergström T, Muhirwa G, Lingh M. Real-time PCR Identification of Agents Causing Diarrhea in Rwandan Children Less Than 5 Years of Age. Pediatr Infect Dis J. 2014;33: 1037–1042. doi:10.1097/INF.0000000000000448

32. Mshana SE, Joloba M, Kakooza A, Kaddu-Mulindwa D. *Campylobacter* spp among Children with acute diarrhea attending Mulago hospital in Kampala - Uganda. Afr Heal Sci. 2009;9: 201–205.

33. Chuma IS, Nonga HE, Mdegela RH, Kazwala RR. Epidemiology and RAPD-PCR typing of thermophilic campylobacters from children under five years and chickens in Morogoro Municipality, Tanzania. BMC Infect Dis. 2016;16. doi:10.1186/s12879-016-2031-z

34. Gosselin KB, Aboud S, McDonald CM, Moyo S, Khavari N, Manji K, et al. Etiology of Diarrhea, Nutritional Outcomes, and Novel Intestinal Biomarkers in Tanzanian infants. JPGN. 2017;64: 104–108. doi:10.1097/MPG.0000000000001323

35. Oketcho R, Nyaruhucha CNM, Taybali S, Karimuribo ED. Influence of enteric bacteria and parasite infection and nutritional status on diarrhoea occurrence in six to 60 month old children admitted at a Regional Hospital in Morogoro, Tanzania. Tanzan J Heal Res. 2012;14: 1–15. doi:10.4314/thrb.v14i2.3

36. Deogratias A-P, Mushi MF, Paterno L, Tappe D, Seni J, Kabymera R, et al. Prevalence and determinants of *Campylobacter* infection among under five children with acute watery diarrhea in Mwanza, North Tanzania. Arch Public Heal. 2014;72. doi:10.1186/2049-3258-72-17

37. Kusiluka LJM, Karimuribo ED, Mdegela RH, Luoga EJ, Munishi PKT, Mlozi MRS, et al. Prevalence and impact of water-borne zoonotic pathogens in water, cattle and humans in selected villages in Dodoma Rural and Bagamoyo districts, Tanzania. Phys Chem Earth. 2005;30: 818–825. doi:10.1016/j.pce.2005.08.025

38. Mdegela RH, Nonga HE, Ngowi HA, Kazwala RR. Prevalence of Thermophilic *Campylobacter* Infections in Humans, Chickens and Crows in Morogoro, Tanzania. J Vet Med B Infect Dis Vet Public Heal. 2006;53: 116–121. doi:10.1111/j.1439-0450.2006.00926.x.

39. Komba EVG, Mdegela RH, Msoffe PLM, Nielsen LN, Ingmer H. Prevalence, Antimicrobial Resistance and Risk Factors for Thermophilic *Campylobacter* Infections in Symptomatic and Asymptomatic Humans in Tanzania. Zoonoses Public Heal. 2015;62: 557–568. doi:10.1111/zph.12185

40. Elfving K, Andersson M, Msellem MI, Welinder-Olsson C, Petzold M, Björkman A, et al. Real-Time PCR Threshold Cycle Cutoffs Help To Identify Agents Causing Acute Childhood Diarrhea in Zanzibar. J Clin Microbiol. 2014;52: 916–923. doi:10.1128/JCM.02697-13

41. Andersson ME, Elfving K, Shakely D, Nilsson S, Msellem M, Trollfors B, et al. Rapid Clearance and Frequent Reinfection With Enteric Pathogens Among Children With Acute Diarrhea in Zanzibar. Clin Infect Dis. 2017;65: 1371–1377. doi:10.1093/cid/cix500

42. Chiyangi H, Muma J., Malama S, Manyahi J, Abade A, Kwenda G, et al. Identification and antimicrobial resistance patterns of bacterial enteropathogens from children aged 0–59 months at the University Teaching Hospital, Lusaka, Zambia: a prospective cross sectional study. BMC Infect Dis. 2017;17. doi:10.1186/s12879-017-2232-0

43. Gwavava C, Chihota VN, Gangaidzo IT, Gumbo T. Dysentery in patients infected with human immunodeficiency virus in Zimbabwe: an emerging role for *Schistosoma mansoni* and *Escherichia coli* O157? Ann Trop Med Parasitol. 2001;95: 509–513. doi:10.1080/00034980120076235

44. Pelkonen T, Dias M, Roine I, Anjos E, Freitas C, Peltola H, et al. Potential Diarrheal Pathogens Common Also in Healthy Children in Angola. Pediatr Infect Dis J. 2018;37: 424–428. doi:10.1097/INF.0000000000001781

45. Zash RM, Shapiro RL, Leidner J, Wester C, McAdam AJ, Hodinka RL, et al. The aetiology of diarrhoea, pneumonia and respiratory colonization of HIV-exposed infants randomized to breast- or formula-feeding. Paediatr Int Child Heal. 2016;36: 189–197. doi:10.1179/2046905515Y.0000000038

46. Rowe JS, Shah SS, Motlhagodi S, Bafana M, Tawanana E, Truong HT, et al. An Epidemiologic Review of Enteropathogens in Gaborone, Botswana: Shifting Patterns of Resistance in an HIV Endemic Region. PLoS One. 2010;5: e10924. doi:10.1371/journal.pone.0010924

47. Pernica JM, Steenhoff AP, Welch H, Mokomane M, Quaye I, Arscott-Mills T, et al. Correlation of Clinical Outcomes With Multiplex Molecular Testing of Stool From Children Admitted to Hospital With Gastroenteritis in Botswana. J Pediatr Infect Dis Soc. 2015;5: 312–318. doi:10.1093/jpids/piv028

48. Alam K, Lastovica AJ, Le Roux E, Hossain MA, Islam MN, Sen SK, et al. Clinical Characteristics and Serotype Distribution of *Campylobacter jejuni* and *Campylobacter coli* Isolated from Diarrhoeic Patients in Dhaka, Bangladesh, and Cape Town, South Africa. Bangladesh J Microbiol. 2006;23: 121–124. doi:10.3329/bjm.v23i2.875

49. Thobela MS, Smith AM, Moonsamy S, du Plessis H, Govender N, Keddy KH. Detection of *Campylobacter* species in stool specimens from patients with symptoms of acute flaccid paralysis in South Africa. J Infect Dev Ctries. 2018;12: 542–549. doi:10.3855/jidc.9795

50. Obi CL, Bessong PO. Diarrhoeagenic bacterial pathogens in HIV-positive patients with diarrhoea in rural communities of Limpopo Province, South Africa. J Heal Popul Nutr. 2002;20: 230–234.

51. Samie A, Obi CL, Barrett LJ, Powell SM, Guerrant RL. Prevalence of *Campylobacter* species, *Helicobacter pylori* and *Arcobacter* species in stool samples from the Venda region, Limpopo, South Africa: Studies using molecular diagnostic methods. J Infect. 2007;54: 558–566. doi:10.1016/j.jinf.2006.10.047

52. Kullin B, Meggersee R, D’Alton J, Galvão B, Rajabally N, Whitelaw A, et al. Prevalence of gastrointestinal pathogenic bacteria in patients with diarrhoea attending Groote Schuur Hospital, Cape Town, South Africa. S Afr Med J. 2015;105: 121–125. doi:10.7196/SAMJ.8654

53. Samie A, Guerrant RL, Barrett L, Bessong PO, Igumbor EO, Obi CL. Prevalence of Intestinal Parasitic and Bacterial Pathogens in Diarrhoeal and Non-diarroeal Human Stools from Vhembe District, South Africa. J Heal Popul Nut. 2009;27: 739–745. doi:10.3329/jhpn.v27i6.4325

54. Samie A, Ramalivhana J, Igumbor EO, Obi CL. Prevalence, Haemolytic and Haemagglutination Activities and Antibiotic Susceptibility Profiles of *Campylobacter* spp. Isolated from Human Diarrhoeal Stools in Vhembe District, South Africa. J Heal Popul Nutr. 2007;25: 406–413.

55. Kalule JB, Smith AM, Vulindhlu M, Tau NP, Nicol MP, Keddy KH, et al. Prevalence and antibiotic susceptibility patterns of enteric bacterial pathogens in human and non-human sources in an urban informal settlement in Cape Town, South Africa. BMC Microbiol. 2019;19. doi:10.1186/s12866-019-1620-6

56. Bonkoungou IJO, Haukka K, Österblad M, Hakanen AJ, Traoré AS, Barro N, et al. Bacterial and viral etiology of childhood diarrhea in Ouagadougou, Burkina Faso. BMC Pediatr. 2013;13. doi:10.1186/1471-2431-13-36

57. Sangaré L, Nikiéma AK, Zimmermann S, Sanou I, Congo-Ouédraogo M, Diabaté A, et al. *Campylobacter* spp. epidemiology and antimicrobial susceptibility in a developing country, Burkina Faso (West Africa). Arf J Cln Exper Microbiol. 2012;13: 106–111. doi:10.4314/ajcem.v13i2.9

58. Sawadogo S, Diarra B, Bisseye C, Compaore TR, Djigma FW, Ouermi D, et al. Molecular Diagnosis of *Shigella*, *Salmonella* and *Campylobacter* by Multiplex Real-Time PCR in Stool Culture Samples in Ouagadougou (Burkina Faso). Sudan J Med Sci. 2017;12: 163–173. doi:10.18502/sjms.v12i3.931

59. Becker SL, Chatigre JK, Gohou J-P, Coulibaly JT, Leuppi R, Polman K, et al. Combined stool-based multiplex PCR and microscopy for enhanced pathogen detection in patients with persistent diarrhoea and asymptomatic controls from Côte d’Ivoire. Clin Microbiol Infect. 2015;21: 591.e1-591.e10. doi:10.1016/j.cmi.2015.02.016

60. Karikari AB, Obiri-Danso K, Frimpong EH, Krogfelt KA. Antibiotic Resistance in *Campylobacter* Isolated from Patients with Gastroenteritis in a Teaching Hospital in Ghana. Open J Med Microbiol. 2017;7: 1–11. doi:10.4236/ojmm.2017.71001

61. Krumkamp R, Sarpong N, Schwarz NG, Adelkofer J, Loag W, Eibach D, et al. Gastrointestinal Infections and Diarrheal Disease in Ghanaian Infants and Children: An Outpatient Case-Control Study. PLoS Negl Trop Dis. 2015;9: e0003568. doi:10.1371/journal.pntd.0003568

62. Steenhard NR, Ørnbjerg N, Mølbak K. Concurrent infections and socioeconomic determinants of geohelminth infection: a community study of schoolchildren in periurban Guinea-Bissau. Trans R Soc Trop Med Hyg. 2009;103: 839–845. doi:10.1016/j.trstmh.2009.05.005

63. Langendorf C, Hello SL, Moumouni A, Gouali M, Mamaty A-A, Grais RF, et al. Enteric Bacterial Pathogens in Children with Diarrhea in Niger: Diversity and Antimicrobial Resistance. PLoS One. 2015;10: e0120275. doi:10.1371/journal.pone.0120275

64. Samuel SO, Aboderin AO, Akanbi AA, Adegboro B, Smith SI, Coker AO. *Campylobacter* enteritis in Ilorin, Nigeria. East Afr Med J. 2006;83: 478–484. doi:10.4314/eamj.v83i09.46770

65. Nwankwo IO, Faleke OO, Salihu MD, Magaji AA, Musa U, Garba J, et al. Detection and viability of *Campylobacter* species isolates from different species of poultry and humans in Sokoto State, Nigeria. Int J One Heal. 2016;2: 19–23. doi:10.14202/ijoh.2016.19-23

66. Obiajuru IOC, Anoule FC, Adogu POU. Emergence of Campylobacteriosis in Orlu, Imo State South Eastern Nigeria, and its Antibiotic Susceptibility. Carib J Sci Tech. 2015;3: 798–804.

67. Hassan-Hanga F, Osisuni K, Ibrahim M. Infectious Diseases in Under-five Children in Kano, North western Nigeria. Int J Infect Dis. 2008;12: e83. doi:10.1016/j.ijid.2008.05.206

68. Smith SI, Otuonye MN, Omonigbehin EA, Nkoth A, Okany CC, Ariyo F, et al. Prevalence of *Campylobacter* species among HIV/AIDS patients in Nigeria. Br J Biomed Sci. 2002;59: 162–163. doi:10.1080/09674845.2002.11978035

69. Gwimi PB, Faleke OO, Salihu MD, Magaji AA, Abubakar MB, Nwankwo IO, et al. Prevalence of *Campylobacter* species in fecal samples of pigs and humans from Zuru Kebbi State, Nigeria. Int J One Heal. 2015;1: 1–5.

70. Aboderin AO, Smith SI, Oyelese AO, Onipede AO, Zailani SB, Coker AO. Role of *Campylobacter jejuni/coli* in diarrhoea in Ile-Ife, Nigeria. East Afr Med J. 2002;79: 423–426.

71. Ohanu ME, Offune J. The Prevalence of *Campylobacter* in Childhood Diarrhoea in Enugu State of Nigeria. J Commun Dis. 2009;41: 117–120.

72. Ibrahim A. *Campylobacter* enteritis in Zaria, North Western Nigeria. Ahmadu Bello University Teaching Hospital. 2014. Available: https://dissertation.npmcn.edu.ng/index.php/FMCPath/article/view/1366

73. Udoh UA. Characterization of *Campylobacter jejuni* as an enteric pathogen in under five children in Jos, Nigeria. Jos University Teaching Hospital. 2013. Available: https://dissertation.npmcn.edu.ng/index.php/FMCPath/article/view/1193/1388

74. Adedapo AE. Prevalence and drug resistance patterns of *Campylobacter* and Listeria species from the stool samples of HIV patients in Ibadan, Nigeria. University of Ibadan. 2018.

75. Ogbomon EO, Whong CMZ, Doko MHI, Magaji SN, Addai TI, Orukotan YF. Prevalence of *Campylobacter* spp. among diarrhoeic HIV-patients in Kaduna, Nigeria. IJAMBR. 2019;7: 70–78. doi:10.33500/ijambr.2019.07.009

76. Sire J-M, Garin B, Chartier L, Fall NK, Tall A, Seck A, et al. Community-acquired infectious diarrhoea in children under 5 years of age in Dakar, Senegal. Paediatr Int Child Heal. 2013;33: 139–144. doi:10.1179/2046905512Y.0000000046

77. Gassama A, Sow PS, Fall F, Camara P, Philippe H, Guyèye-N’diaye A, et al. Ordinary and opportunistic enteropathogens associated with diarrhea in senegalese adults in relation to Human Immunodeficiency Virus serostatus. Int J Infect Dis. 2001;5: 192–198. doi:10.1016/S1201-9712(01)90069-4

78. Hoy D, Brooks P, Woolf A, Blyth F, March L, Bain C, et al. Assessing risk of bias in prevalence studies: Modification of an existing tool and evidence of interrater agreement. J Clin Epidemiol. 2012;65: 934–939. doi:10.1016/j.jclinepi.2011.11.014
