# Supplementary material for: A systematic review and meta-analysis reveal that Campylobacter spp. and antibiotic resistance are widespread in humans in sub-Saharan Africa
Source: PLoS One. 2021 Jan 27;16(1):e0245951. doi: 10.1371/journal.pone.0245951 (PMC7840040; doi:10.1371/journal.pone.0245951)
Supplement: S3 Table — (DOCX) [file pone.0245951.s005.docx]

**A systematic review and meta-analysis reveal that *Campylobacter* spp. and antibiotic resistance are widespread in humans in sub-Saharan Africa**

**S3 Table. Detailed information about *Campylobacter* spp. studies in humans from sub-Saharan Africa from 2000 to 2020.**

| **Nr** | **Citation** | **DATABASE SOURCE** | **Country (study site: major to minor)** | **Time-frame** | **Study design** | **Type of samples** | **Diagnostic Method** | **Nr individuals** | **Prevalence** | **95% CI** | **Genus and species** |
| --- | --- | --- | --- | --- | --- | --- | --- | --- | --- | --- | --- |
| **Eastern Africa** | | | | | | | | | | | |
|  | [1] | AJOL | Ethiopia (Jimma University Specialized Hospital) | September 2002 - June 2003 | **Cross-sectional**  Inclusion: children <15 years with diarrhea or dysentery.  Exclusion: NR | Fresh stool | Culture: *Campylobacter* agar plates; Microscopy: gram stain;  Biochemical tests: oxidase and catalase | 430 | 11.6%, n=50 | 8.8%-15.0% | *Campylobacter* spp. |
|  | [2] | AJOL | Ethiopia (Dembia District: Kolla Diba town) | April - May 1998 | **Cross-sectional**  Inclusion: children <15 years with diarrhea residing in the villages  Exclusion: NR | Stool | Culture: *Campylobacter* agar plates; Microscopy: gram stain | 153 | 10.5%, n=16 | 6.1%-16.4% | *Campylobacter* spp. |
|  | [3] | PUBMED | Ethiopia (Jimma: Jimma University Specialized Hospital (JUSH) and Jimma Health Center [JHC]) | July - October 2012 | **Cross-sectional**  Inclusion: children < 5 years with diarrhea  Exclusion: NR | Stool | Culture: blood *free Campy-*  *lobacter* selective agar base containing CAT selective supplement; Microscopy: Gram stain; Motility test; Biochemical test: hippurate hydrolysis;  Susceptibility to cephalothin and nalidixic acid | 227 | 16.7%, n=38 | 12.1%-22.3% | *C. jejuni* (11.9%, n=27)  *C. coli* (3.5%, n=8)  *C. lari* (1.3%, n=3) |
|  | [4] | PUBMED | Ethiopia (Bahir Dar: Felege Hiwot Hospital) | October 2007- April 2008 | **Cross-sectional**  Inclusion: patients with enteric signs (including at least diarrhea, stomach cramps, nausea, vomiting and fever)  Exclusion: NR | Stool | Culture: mCCDA; Microscopy: Gram stain; Biochemical tests: oxidase, catalase, hippurate hydrolysis and H_2_S production. | 210 | 8%, n=17 | 4.8%-12.6% | *C. jejuni* (7.6%, n=16)  *C. coli* (0.5%, n=1) |
|  | [5] | PUBMED and AJOL | Ethiopia (Hawassa: Adare Hospital and Millennium Health Center) | June 6 - October 28, 2011 | **Cross-sectional**  Inclusion: children <5 years with diarrhea  Exclusion: children who had taken antibiotic within 7 days before data collection; > 5 years old; and whose parents/guardians were not voluntary. | Stool and rectal swab | Culture: CCDA;  Microscopy: Gram stain  Biochemical tests: oxidase and catalase | 158 | 12.7%, n=20 | 7.9%-18.9% | *Campylobacter* spp. |
|  | [6] | PUBMED and CINAHL | Ethiopia (University of Gondar Teaching Hospital) | October, 2011 - March, 2012 | **Cross-sectional**  Inclusion: <5 years old children with diarrhea  Exclusion: <5 years old children with diarrhea who had treatment with antibiotics in the last 5 days | Stool | Culture: *Campylobacter* Agar Base (Karmali) supplemented;  Microscopy: gram stain; Biochemical tests: oxidase, catalase. | 285 | 15.4%, n=44 | 11.5%-20.2% | *C. jejuni/coli* (14.0%, n=40)  Other *Campylobacter* spp. (1.4%, n=4) |
|  | [7] | PUBMED | Ethiopia (Hawassa city: Hawassa University Comprehensive Specialized Hospital [HUCSH]) | February - May 2016 | **Cross-sectional**  Inclusion: HIV-infected patients with signs and symptoms of gastrointestinal tract disease.  Exclusion: Patients <18 years, or who could not provide a stool sample or who had taken antimicrobial treatment (except trimethoprim-sulphamethoxazole [SXT] prophylaxis) within 2 weeks. | Stool | Culture: blood free *Campylobacter* selective agar with supplement;  Biochemical tests | 215  Diarrheic  102  Non-diarrheic  113 | 6.04%, n=13  Diarrheic  10.8%, n=11  Non-diarrheic  1.8% n=2 | 3.3%-10.1%  Diarrheic  5.5%-18.5%  Non-diarrheic  0.2%-6.2% | *Campylobacter* spp. |
|  | [8] | PUBMED | Kenya (Asembo: a mission clinic and a hospitall) | May 1997 -April 1998 | **Cross-sectional**  Inclusion: Patients with diarrhea (a) and without (b)  Exclusion: NR | Stool or rectal swabs | Culture (Medium NR) | 829  a.729  b.100 | 9.8%, n=81  a.10%, n=73  b.8%, n=8 | 7.8%-12.0%  a.7.9%-12.4%  b.3.5%-15.2% | a. and b*. Campylobacter* spp.  *a. C. jejuni* (7.8%, n=57)  *a. C. coli* (0.8%, n= 6)  *a. C. lari* (0.8%, n=6)  *a. C. jejuni/coli* (0.5%, n=4) |
|  | [9] | Sciencedirect | Kenya (Nairobi: Kibera, Tabitha Medical Clinic) | November, 1, 2008- December, 31, 2011 | **Cross-sectional**  Inclusion: Persons without current or previous 2 weeks history of fever, respiratory or diarrheal symptoms  Exclusion: NR | Stool | Culture (Medium NR) | 384 | 17.3% *C. jejuni*  4.3% *C. coli* | - | *C. jejuni* (17.3%, n=66?)  *C. coli* (4.3%, n=17?) |
|  | [10] | Sciencedirect | Kenya (Kisum: outpatient clinic of the Nyanza  Provincial General Hospital) | 1997 - 2001 | **Nested cohort**  Inclusion: Cases: children < 2 years with diarrhea; controls: children without diarrhea  Exclusion: with indeterminate HIV status. | Stool  or rectal swabs | Culture (Medium NR) | Cases  630  Controls  91 | Cases  20.8%, n=131  Controls  16.5%, n=15 | Cases  17.7%-24.2%  Controls  9.5%-25.7% | *Campylobacter* spp. |
|  | [11] | PUBMED | Kenya (Nyanza Province, Siaya District, Asembo Bay: 3 rural clinics and an outpatient clinic) | May 1, 1997 -September 30, 2001 | **Case-control**  Inclusion: Cases: persons with bloody diarrhea; controls: persons with non-acute medical conditions  Exclusion: excluded as controls persons who reported any form of diarrhea or any other gastrointestinal symptoms within 14 days of enrollment. | Stool | Culture (Medium NR) | 451  Cases  97 | 7%, n=33  Cases  6%, n=6 | 5.1%-10.1%  Cases  2.3%-13.0% | *C. jejuni* (5%, n=21)  *C. coli* (1%, n=6)  *C. jejuni*/*C. coli* (<1%, n=1)  Non-*jejuni*, Non-*coli Campylobacter* (1%, n=5)  Cases  *C. jejuni* (3%, n=3)  *C. coli* (1%, n=1)  C. *jejuni*/*C. coli* (0%, n=0)  Non-*jejuni*, Non-*coli Campylobacter* (2%, n=2) |
|  | [12] | PUBMED | Kenya (Kisii Provincial and Homa Bay District Hospital) | November 2011 - October 2013 | **Cross-sectional**  Inclusion: children aged 6 months to 15 years with acute diarrhea  Exclusion: unaccompanied by a biological parent or legal guardian, unable to provide a stool sample or rectal swab, or if the primary caregiver elected not to receive HIV counseling on behalf of the child. | Stool and rectal swabs | Culture (cefoperazone vancomycin amphotericin [CVA] agar) | 1076 | 6.3%, n=68 | 4.9%-7.9% | *C. jejuni* (4.2%, n=45)  Other *Campylobacter* spp. (2.1%, n=23) |
|  | [13] | PUBMED | Kenya (Nyanza province: Kisii Provincial Hospital and Homa Bay District Hospital) | November 2011 - June 2014 | **Cross-sectional**  Inclusion: HIV-uninfected children 6 months to 5 years with diarrhea.  Exclusion: if they were not accompanied by a legal guardian or biological parent, if study staff were unable to collect a stool sample or rectal swab or if the primary caregiver refused HIV testing on behalf of the child. | Stool and rectal swabs | Culture  and serotyping methods. | 1363 | 7.4%, n=101 | 6.1%-8.9% | *Campylobacter* spp. |
|  | [14] | Google Scholar | Kenya (Nandi County: Kapsabet County Hospital) | NR | **Cross-sectional**  Inclusion: children <5 years with diarrhea  Exclusion: NR | Stool | Standard bacteriological methods (not specified); Biochemical tests: catalase, oxidase, hippurate hydrolysis, H_2_S production;  Susceptibility to nalidixic acid | 139 | 39.1% | - | *C. jejuni* |
|  | [15] | PUBMED | Kenya (Nyanza Province: Bondo and Siaya District Hospitals) | May 23 2005 - May 22, 2007 | **Cohort**  Inclusion: children <5 years old with diarrhea  Exclusion: NR | Stool and/or rectal swabs | Culture: *Campylobacter* selective media;  Biochemical test: hippurate hydrolysis | 1146  Died  107  Survived  1039 | 5%, n=57  Died  5%, n=5  Survived  5%, n=52 | 3.8%-6.4%  Died  1.5%-10.6%  Survived  3.8%-6.5% | *C. jejuni* (80%, (3.7%?) n=4? of those who died; 83%, (4.1%?) n=43? of those who survived)  Other *Campylobacter* spp. |
|  | [16] | PUBMED and AJOL | Kenya (Nyanza Province, Kisumu and Asembo Bay: Nyanza Provincial Hospital and 4 clinics) | 1997-2003 | **Cross-sectional**  Inclusion: children <5 years with diarrhea  Exclusion: NR | Stool | Culture (Medium NR) and serotyping NR | Urban  1303  Rural  1247 | Urban  17%, n=224  Rural  15%, n=193 | Urban  15.2%-19.4%  Rural  13.5%-17.6% | *Campylobacter* spp. |
|  | [17] | PUBMED | Kenya (Nyanza Province, Asembo Bay: 3 clinics and one outpatient clinic of a mission hospital) | May 1997 - April 2003 | **Cross-sectional**  Inclusion: Persons with diarrhea  Exclusion: NR | Stool or rectal swab | Culture (Medium NR) | 3445 | 8.5%, n=294 | 7.6%-9.5% | *C. jejuni* (2.8%, n=97)  *C. coli* (0.6%, n=22) |
|  | [18] | PUBMED | Kenya (Nyanza Province, Kericho District Hospital and Kisumu District Hospital) | September 2009 - September 2011 | **Case-control**  Inclusion: Cases: patients with acute diarrhea; Controls Age-matched with no episodes of diarrhea within the 2 weeks  Exclusion: NR | Stool | Culture (CVA plates)  Phenotypic analyses (not specified) | **Kericho** | **Kericho** | **Kericho** | *Campylobacter* spp. |
|  |  |  |  |  |  |  |  | 386 | 0.8%, n=3 | 0.2%-2.3% |  |
|  |  |  |  |  |  |  |  | Cases | Cases | Cases |  |
|  |  |  |  |  |  |  |  | 193 | 1%, n=2 | 0.1%-3.7% |  |
|  |  |  |  |  |  |  |  | Controls | Controls | Controls |  |
|  |  |  |  |  |  |  |  | 193 | 0.5%, n=1 | 0.0%-2.9% |  |
|  |  |  |  |  |  |  |  | **Kisumu** | **Kisumu** | **Kisumu** |  |
|  |  |  |  |  |  |  |  | 478 | 1.9%, n=9 | 0.9%-3.6% |  |
|  |  |  |  |  |  |  |  | Cases | Cases | Cases |  |
|  |  |  |  |  |  |  |  | 239 | 2.1%, n=5 | 0.7%-4.8% |  |
|  |  |  |  |  |  |  |  | Controls | Controls | Controls |  |
|  |  |  |  |  |  |  |  | 239 | 1.7%, n=4 | 0.5%-4.2% |  |
|  | [19] | PUBMED | Kenya (Nyanza Province: St Elizabeth Mission Hospital) | November 4, 2009 - February 4, 2011 | **Case-control**  Inclusion: Cases: children 0–59 months with moderate to severe diarrhea. Controls: children without diarrhea matched to individual cases by age, sex, and residence  Exclusion: NR | Stool | Culture: Campy blood agar plate;  Microscopy: Gram stain;  Biochemical tests: oxidase, catalase, sodium hippurate. | 146  Cases  73  Controls  73 | 22.6%, n=33  Cases  19.2%, n=14  Controls  26%, n=19 | 16.1%-30.3%  Cases  10.9%-30.1%  Controls  16.5%-37.6% | Cases:  *C. jejuni* (12%, n=9)  *C. coli* (7%, n=5)  Controls:  *C. jejuni* (16%, n=12)  *C. coli* (10%, n=7) |
|  | [20] | Google Scholar | Kenya (Nairobi County: 11 primary schools located at Kibera informal settlement) | July, 2015 | **Cross-sectional**  Inclusion: asymptomatic children  Exclusion: NR | Stool | Culture: mCCDA with supplement; Biochemical tests: oxidase and peroxidase breakdown; Multiplex PCR | 580 | 18%, n=106 | 15.2%-21.7% | *C. jejuni*  (7.6%, n=44)  *C. coli* (4.8%, n=28)  *C. lari* (1.9%, n=11)  Other *Campylobacter* spp. (4.0%, n=23) |
|  | [21] | PUBMED, Google Scholar and CINAHL | Madagascar (Moramanga, Two villages) | January 2010 -May 31, 2012 | **Prospective cohort**  Inclusion: children <24 months with (a) and without (b) diarrhea  Exclusion: NR | Stool | Culture on selective agar plates (Karmali); Campy dry spot kit, a haemagglutination test. Multiplex PCR | 3424 samples (508 children)  a. 459  b. 2965 | 9.3%, n=319  a.  8.9%, n= 41  b.  9.4%, n=278 | 8.4%-10.3%  a.  6.5%-11.9%  b.  8.4%-10.5% | *C. jejuni* (5.5%, n=190)  *C. coli* (1.9%, n=64)  Other *Campylobacter* spp. (0.5%, n=17)  *it is not known to which group the isolates belong. |
|  | [22] | PUBMED | Madagascar (Moramanga and Antananarivo: 2 hospitals) | November 2011 - January 2014 | **Matched case-control**  Inclusion: Cases: children aged 0 to 59 months with severe diarrhea. Controls: matched with the cases for age, sex and residence, good health and had not suffered from diarrhea or used antibiotics in 7 days before the survey  Exclusion: NR | Stool | Culture (Karmali agar);  Haemagglutination test kit (Campy dry spot, from Oxoid, UK) | 398  Cases  199  Controls 199 | Cases  1%, n=2  Controls  0.5%, n=1 | Cases  0.1%-3.6%  Controls  0.0%-2.8% | *Campylobacter* spp. |
|  | [23]  Part III | Google Scholar | Madagascar (14 locations; Communities) | February 2008 – May 2009 | **Case-control study**  Inclusion: Cases: children <5 years with acute diarrhea and of different family origin who did not take antibiotics. Controls: <5 years old without diarrhea.  Exclusion: hospitalized patients | Stool | Culture (Karmali's medium);  Confirmation by a haemagglutination test ("Campy dry spot") | 2692  Cases  2196 Controls  496 | 9.5%, n=256  Cases  9.5%, n=209  Controls  9.5%, n=47 | 8.4%-10.7%  Cases  8.3%-10.8%  Controls  7.1%-12.4% | Cases  *C. jejuni* (7.2%, n=159)  *C. coli* (2.3%, n=50)  Controls  *C. jejuni* (7.3%, n= 36)  *C. coli* (2.2%, n=11) |
|  | [24] | PUBMED and Google Scholar | Malawi (Blantyre: Queen Elizabeth Central Hospital) | 1997–2007 | **Prospective case-control**  Inclusion: Cases: children <5 years with diarrhea. Controls: without diarrhea  Exclusion: NR | Stool | Real-time PCR | 2448  Cases  1941  Controls 507 | Cases  21%, n=415  Controls  14%, n=69 | Cases  19.6%-23.3%  Controls  10.8%-16.9% | Cases  *C. jejuni* (85% (18.2%), n=353?)  *C. coli* (15%, (3.2%) n=62?)  Controls  Unclear |
|  | [25] | Sciencedirect | Malawi (Blantyre: NRU Moyo House of Queen Elizabeth Central Hospital) | January - July 2013 | **Cohort**  Inclusion: severely malnourished children, aged 8-59 months.  Exclusion: confirmed or clinically suspected malaria, tuberculosis or children received treatment; with insufficient or missing stool samples for analysis | Stool | Multiplex PCR | 47 | Prevalence at admission  34%, n=16  After clinical stabilization  2%, n=1 | Prevalence at admission  20.9%-49.3%  After clinical stabilization  0.0%-11.3% | *C. jejuni/ coli* |
|  | [26] | PUBMED | Malawi (Blantyre: Queen Elizabeth Central Hospital) | January - July 2013 | **Cohort**  Inclusion: children aged 6–60 months with severe acute malnutrition (SAM)  Exclusion: HIV positive or exposed  readmitted to hospital for SAM within the past year; packed cell volume of <15%; severe hemodynamic instability; unknown HIV status; severe neurologic symptoms; confirmed or clinically suspected tuberculosis, malaria  or insufficient serum for analyses. | Stool | Multiplex PCR | 64  Recovered  53  Death  11 | 30%, n=19  Recovery  30%, n=16  Death  27%, n=3 | 18.9%-42.4% Recovery  18.3%-44.3%  Death  6.0%-61.0% | *C. jejuni/ coli* |
|  | [27] | PUBMED | Mozambique (Maputo: Manhiça District, Manhiça District Hospital) | September 2000 -September 2001 | **Cross-sectional**  Inclusion: hospitalized children < 5 years of age presenting diarrhea in their diagnosis  Exclusion: NR. | Stool | Culture (medium NR);  Microscopy: Gram stain;  Biochemical test: oxidase test | 529 | 1.7%, n=9 | 0.8%-3.2% | *Campylobacter* spp. |
|  | [28] | Other sources | Mozambique (Maputo: 17 neighborhoods) | February 2015 - February 2016 | **Cross-sectional**  Inclusion: children aged 1–48 months, independent of reported symptoms; informed consent; residing in compounds meeting inclusion criteria  Exclusion: NR | Stool | Multiplex RT-PCR | 759 | 8%, n=61 | 6%–10% | *Campylobacter* spp. |
|  | [29] | Other sources | Mozambique (Maputo, Manhiça District: health facilities) | December 2007 - October 2011 | **Case-control**  Inclusion: Cases: children aged 0–59 months with moderate-to-severe diarrhea. Controls: children with no story of diarrhea in the previous 7 days matched by age and gender.  Exclusion: NR | Stool | Culture: Campy blood agar;  Microscopy: Gram stain  Biochemical tests: Oxidase, catalase, hippurate hydrolysis | Cases  784  Controls  1545 | Cases  4.2%, n=33  Controls  Unclear | Cases  2.9%-5.9%  Controls  Unclear | *Campylobacter* spp. |
|  | [30] | Sciencedirect | Rwanda (Kigali and Butare: 3 health centres) | November 2009 - June 2012 | **Cross-sectional**  Inclusion: age ≤5.0 years and diarrhea with duration of <96 h with or without vomiting or fever.  Exclusion: non-enteric acute infections, severe malnutrition and AIDS | Stool and rectal swab | Real-time PCR | 880 | 16.7%, n=147 | 14.3%-19.3% | *Campylobacter* spp. |
|  | [31] | PUBMED | Rwanda (2 health centres, 2 district hospitals and 2 university hospitals) | 2011–2012 | **Case-control**  Inclusion: Cases: age ≤5.0 years and diarrhea with a duration of <96 hours (with or without vomiting or fever). Controls: healthy children below 5 years, living in the same geographic area as the patients, without any episode of diarrhea within 2 weeks  Exclusion: NR | Rectal swab | Real-time PCR | 706  Cases  544  Controls  162 | 15.7%, n= 111  Cases  15%, n=81  Controls  19%, n=30 | 13.1%-18.6%  Cases  12.0%-18.2%  Controls  12.9%-25.4% | *Campylobacter* spp. |
|  | [32] | AJOL and Google Scholar | Uganda (Kampala, Mulago Hospital) | July - October 2005 | **Cross-sectional**  Inclusion: children from 1 month to 5 years with acute diarrhea; written signed consent from the parent/caretaker.  Exclusion: children with > 5 years or <1 month; with persistent diarrhea; no written or signed consent from parent/caretakers. | Stool | Culture: blood free CCDA and blood contained media selective for *Campylobacter*; Microscopy: gram stain; Biochemical tests:  catalase test, oxidase test, hippurate hydrolysis;  Susceptibility to nalidixic acid and cephalothin. | 226 | 9.3%, n=21 | 5.8%-13.9% | *C. jejuni* (7.5%, n= 17)  *C. coli* (0.4%, n= 1)  *C. lari* (0.9%, n= 2)  *C. jejuni/coli* (0.4%, n=1) |
|  | [33] | PUBMED and CINAHL | Tanzania (Morogoro Municipality: Morogoro Regional and Mazimbu Hospitals, Mafiga, Madizini, Usangi and Upendo health facilities) | December 2006 - May 2007 | **Cross-sectional**  Inclusion: Children <5 years of age with (a) or without diarrhea (b).  Exclusion: Children admitted, hospitalized and those under antibiotic therapy | Stool | Culture (*Campylobacter* Enrichment Broth, mCCDA supplemented; Microscopy Gram’s stain; Motility test;  Biochemical tests: catalase, oxidase, nitrate reduction, hippurate hydrolysis.  RAPD-PCR | 268  a.  78  b.  190 | 19%, n=51  a.  12.8%, n=10  b.  21.6%, n=41 | 14.5%-24.3%  a.  6.3%-22.3%  b.  16.0%-28.1% | *C. jejuni* (14.9%, n=40)  *C. coli* (3.7%, n=10)  Unidentified (0.4%, n=1)  *it is not known to which group the isolates belong. |
|  | [34] | PUBMED | Tanzania (Dar es Salaam: Clinic) | January 2009 -  January 2011 | **Cross-sectional**  Inclusion: infants (<1 year) with acute diarrhea from HIV - mothers  Exclusion: infants of multiple births and with congenital anomalies or other conditions | Stool | PCR | 123 | 2.4%, n=3 | 0.5%-7.0% | *C. jejuni/coli* |
|  | [35] | AJOL and PUBMED | Tanzania (Morogoro, Morogoro Regional Hospital) | January - September 2011 | **Case-control**  Inclusion: 6-60 months old children with diarrhea (cases), without diarrhea (controls)  Exclusion: (for controls) those who had had diarrhea within the last two consecutive weeks | Stool | Culture (modified CCDA-Preston with supplement)  Microscopy: Gram staining  Biochemical test: hippurate test | 303  Case  151  Control  152 | 1.7%, n=5  Case  3.3%, n=5  Control  0%, n=0 | 0.54%-3.81%  Case  1.08%-7.56%  Control  0.00%-2.40% | *C. jejuni* |
|  | [36] | PUBMED, Google Scholar and CINAHL | Tanzania (Mwanza city, BMC and Sekou-Toure hospital) | October 2012 - April 2013 | **Cross-sectional**  Inclusion: Children aged between 1 to 60 months with acute watery diarrhea  Exclusion: NR | Stool | Culture (Preston selective agar)  Microscopy | 300 | 9.7%, n=29 | 6.57%-13.59% | *Campylobacter* spp. |
|  | [37] | Sciencedirect | Tanzania (Dodoma Rural and Bagamoyo districts: Mundemu, Chamwino and Chalinze health centres) | 2003-2004 | **Cross-sectional**  Inclusion: volunteers who visited the health centers  Exclusion: NR | Stool | Culture: nutrient  broth enriched with Preston supplement, mCCDA; Microscopy: gram stain; Biochemical tests: catalase, oxidase, nitrate, hippurate hydrolysis; Susceptibility to cephalothin. | 484 | 1.9%, n=9 | 0.9%-3.5% | *C. jejuni* |
|  | [38] | PUBMED and Google scholar | Tanzania (Morogoro: Morogoro regional hospital, Sokoine University of Agriculture dispensary and Upendo medical laboratory) | January 2003 - December 2004 | **Cross-sectional**  Inclusion: patients presenting enteric signs including at least diarrhea, stomach cramps, nausea, vomiting and fever.  Exclusion: NR | Stool | Culture: nutrient broth with Preston supplements, mCCDA, subculture on blood agar; Microscopy: Gram stain; Motility test; Biochemical tests: catalase, oxidase, nitrate reduction, hippurate hydrolysis; Susceptibility to cephalothin;  PCR | 632 | 9.3%, n=59 | 7.2%-11.9% | *C. jejuni* (9.0%, n=57)  *C. coli* (0.3%, n=2) |
|  | [39] | PUBMED and Google Scholar | Tanzania (Morogoro: Morogoro regional hospital, Sokoine University of Agriculture dispensary and Upendo medical laboratory) | December 2011 - April 2012 | **Cross-sectional**  Inclusion: individuals with (a) and without enteric complaints (b)  Exclusion: NR | Stool | Culture: antibiotic-free blood agar; Microscopy:  Gram stain; Motility test;  Biochemical test: sodium hippurate hydrolysis; PCR | 1195  a.  898  b.  297 | 11.4%, n=136  a.  12.9%, n=116  b.  6.7%, n=20 | 9.6%-13.3%  a.  10.8%-15.3%  b.  4.2%-10.2% | *C. jejuni* (9.6% n=115)  *C. coli* (1.8%, n=21) |
|  | [40] | PUBMED | Tanzania (Zanzibar: (Kivunge Primary Health Care Centre) | April - July 2011 | **Case-control**  Inclusion: Cases: children 2 to 59 months with fever and diarrhea; Controls: asymptomatic matched for living area and sampling time period  Exclusion: children with signs of severe disease | Rectal swab | RT-PCR | 330  Cases  165  Controls  165 | 33.9%, n=112  Cases  35%, n=58  Controls  33%, n=54 | 28.8%-39.3%  Cases  27.9%-43.0%  Controls  25.6%-40.5% | *Campylobacter* spp. |
|  | [41] | PUBMED | Tanzania (Zanzibar: (Kivunge Primary Health Care Centre) | April–July 2011 | **Cohort**  Inclusion: children aged 2–59 months with diarrhea  Exclusion: NR | Rectal swab | RT-PCR | 127 | Baseline  34%, n=43    Follow up  30%, n=38 | Baseline  25.7%-42.8%  Follow up  22.1%-38.7% | *Campylobacter* spp. |
|  | [42] | PUBMED | Zambia (Lusaka: University Teaching Hospital) | December 2015 - April 2016 | **Cross-sectional**  Inclusion: children aged 0–59 months with diarrhea  Exclusion: NR | Stool | Culture\|: mCCDA  Biochemical tests (API-Campy Strips) | 85 | 3.5%, n=3 | 0.7%-10.0% | *C. jejuni* |
|  | [43] | PUBMED | Zimbabwe (Harare: Harare Central Hospitals,  Parirenyatwa General Hospital, Beatrice Road Infectious Diseases Hospital and Wilkins Infectious Diseases Hospital) | December 1999 - March 2000 | **Cross-sectional**  Inclusion: patients ≥ 18 years, with  bloody diarrhea  Exclusion: patients who had undergone intestinal surgery or had known inflammatory bowel disease | a. Blood  b. Stool | Culture: Kirrow’s agar | 40  HIV +  25  HIV –  15 | a. Blood  0%  b. Stool  1%, n=2  HIV +  4%, n=1  HIV –  7%, n=1 | a. Blood  0.0%-8.8%  b. Stool  6.2%-15.0%  HIV +  0.1%-20.4%  HIV –  0.2%-31.9% | *C. jejuni* |
| **Middle Africa** | | | | | | | | | | | |
|  | [44] | PUBMED | Angola (Luanda: Pediatric Hospital of Luanda) | December 25, 2013 - October 5, 2014 | **Case-control**  Inclusion: Cases: children <5 years of age whose diarrhea had lasted less than 2 weeks; Controls: children with no diarrhea  Exclusion: NR | Stool | Multiplex RT-PCR | 194  Cases  98  Controls  96 | 15%, n=29  Cases  23%, n=23  Controls  6%, n=6 | 10.1%-20.6%  Cases  15.5%-33.1%  Controls  2.3%-13.1% | *Campylobacter* spp. |
| **Southern Africa** | | | | | | | | | | | |
|  | [45] | PUBMED | Botswana (4 hospitals) | March 2001 - October 2003 | **Cross-sectional**  Inclusion: infants (<1 year) born to HIV-infected mothers with acute diarrhea  Exclusion: NR | Stool | Culture: *Campylobacter* media  Microscopy: Gram stain. | 300 | 7.3%, n=22 | 4.7%-10.9% | *Campylobacter* spp. |
|  | [46] | PUBMED | Botswana  (Gaborone: Princess Marina Hospital, clinics and smaller hospitals) | February 1, 2003 - July 31, 2008 | **Retrospective, cross-sectional**  Inclusion: Stool samples received from the study locations  Exclusion: patient without gastroenteritis | Stool | Culture and Microscopy (not specified) | 4485 | 0.5%, n= 22 | 0.3%-0.7% | *Campylobacter* spp. |
|  | [47] | PUBMED | Botswana (Gaborone: Princess Marina Hospital and Nyangabgwe Referral Hospital) | May 2011 -April 2013 | **Prospective cohort**  Inclusion: inpatients < 13 years with diarrhea present for less than 14 days and had at least 3 loose stools, or a single loose stool with at least 2 episodes of emesis, in any 24-hour period prior to enrollment.  Exclusion: children who developed diarrhea >48 hours after admission or who had been discharged within 7 days of diarrhea. | Stool | PCR: Gastrointestinal Pathogen Panel (GPP) assay | 671 | 14%, n= 95 | 11.6%-17.0% | *Campylobacter* spp. |
|  | [48] | Google Scholar | South Africa (Cape Town: Red Cross Children’s Hospital) and other country outside sub-Saharan Africa (not reported here) | 2000-2002 | **Cross-sectional**  Inclusion: diarrhea patients of all age groups  Exclusion: NR | Stool | Culture: antibiotic-free tryptose blood agar and other tests (not specified); Serotyping. | 5635 | 16.3%, n= 916 | 15.3%-17.3% | *C. jejuni* subsp. *jejuni* (5.6%, n=316)  biotypes 1 & 2  *C. jejuni* subsp. *doylei* (1.6%, n=91)  *C. concisus* (4.2%, n=234)  *C. upsaliensis* (4.0%, n=226)  *C. coli* (0.6%, n=32)  *C. hyointestinalis* (0.2%, n=13)  *C. fetus* subsp. *fetus* (0.02%, n=1)  *C. lari* (0.02%, n=1)  *C. curvus* (0.02%, n=1)  *C. sputorum* biovar. s*putorum* (0.02%, n=1) |
|  | [49] | Google scholar | South Africa (Regional Polio Reference Laboratory) | October 2014 - December 2015 | **Cross-sectional**  Inclusion: stool specimens from patients with acute flaccid paralysis, that were negative for polio and non-polio enteroviruses (NPENT),  Exclusion: positive samples for polio virus and NPENT | Stool | Multiplex qPCR | 512 | 12%, n=62 | 9.4%-15.3% | *C. jejuni* (9.4%, n= 48)  *C. coli* (2.3%, n=12)  *C. jejuni* and *C. coli.* (0.4%, n=2) |
|  | [50] | PUBMED | South Africa (Limpopo Province: Clinics) | March 2001 -February  2002 | **Cross-sectional**  Inclusion: HIV positive patients with chronic diarrhea  Exclusion: NR | Stool | Culture: Skirrow’s and Butzler’s media; Biochemical tests: hippurate and indoxyl  acetate hydrolysis. | 60 | 20.0%, n=12 | 10.8%-32.3% | *C. jejuni* (13.3%, n=8)  *C. coli* (6.7%, n=4) |
|  | [51] | PUBMED and Sciencedirect | South Africa (Limpopo, Venda region: 3 hospitals and 2 primary schools) | November 2004 - May 2005 | **Cross-sectional**  Inclusion: patients with gastrointestinal complaints or with diarrhea; primary school children regardless of symptoms. (a) diarrhea; (b) no diarrhea; HIV positive and negative.  Exclusion: NR | Stool | Culture: mCCDA; haemagglutination kit ‘‘Campy Dry Spot’’  PCR | 322  a.  162  b.  160 | 19.9%, n=64  a.  32.0%, n=52  b.  7.5%, n=12 | 15.7%-24.7%  a.  25.0%-39.9%  b.  3.9%-12.7% | *C. jejuni* (10.2%, n=33)  *C. coli* (6.5%, n=21)  *C. concisus* (3.1%, n=10) |
|  | [52] | PUBMED and AJOL | South Africa (Cape Town: Groote Schuur Hospital) | March 2012 - March 2013 | **Cross-sectional**  Inclusion: in- and outpatients with diarrhea  Exclusion: patients <16 years | Stool | PCR | 139 | 0%, n=0 | 0.0%-2.6% | *Campylobacter* spp. |
|  | [53] | PUBMED | South Africa (Vhembe district: 3 hospitals and 2 primary schools) | October 2003 - April 2005 | **Cross-sectional**  Inclusion: children from primary schools and patients attending hospitals with (a) or without (b) diarrheic stools  Exclusion: NR | Stool | Culture (not described)  Confirmation by the haemagglutination test-kit ‘Campy dry spot’ | 823  Hospitals  528  a.  269  b.  259  School  295  a.  39  b.  256 | 11.9%, n=98  Hospitals  16.8%, n=89  a.  24.9%, n=67  b.  8.5%, n=22  School  3.1%, n=9  a.  12.8%, n=5  b.  1.6%, n=4 | 9.8%-14.3%  Hospitals  13.8%-20.3%  a.  19.9%-30.5%  b.  5.4%-12.6%  School  1.4%-5.7%  a.  4.3%-27.4%  b.  0.4%-4.0% | *Campylobacter* spp. |
|  | [54] | PIBMED and Google Scholar | South Africa (Vhembe District: Elim, Tshilidzini, Siloam and Vhufuli Hospitals) | November 2004 - May 2005 | **Cross-sectional**  Inclusion: patients with diarrhea  Exclusion: NR | Stool | Culture: Columbia blood agar plate supplemented/ Skirrow’s media/mCCDA; Microscopy: gram stain; Motility test;  Biochemical tests: oxidase, catalase,  haemagglutination kit ‘Campy Dry Spot’; haemolytic activity; β-lactamase production; PCR | 565 | 20.4%, n=115 | 17.1%-23.9% | *C. jejuni* (17.3%, n=98)  *C. coli.* (3.0%, n=17) |
|  | [55] | PUBMED | South Africa (Cape Town: Nyanga urban informal settlement - Community Health Center) | October 2015 – April 2016 | **Cross-sectional**  Inclusion: children < 12 years of age with diarrhea  Exclusion: NR | Stool | Culture: mCCDA | 66 | 5%, n=3 | 0.9%-12.7% | *C. jejuni* |
| **Western Africa** | | | | | | | | | | | |
|  | [56] | PUBMED | Burkina Faso (Ouagadougou: Centre Médical avec Antenne Chirugicale) | January 2009 - January 2010 | **Case-control**  Inclusion: Case: children  < 5 years with acute diarrhea; Control: children with no history of diarrhea for at least 21 days prior to visiting  Exclusion: NR | Stool | Culture: mCCDA | Cases  283  Controls  60 | Cases  2%, n=5  Controls  0%, n=0 | Cases  0.6%-4.1%  Controls  0.0%-6.0% | Cases  *C. jejuni* (1.1%, n=3)  *C. coli* (0.7%, n=2)  Controls  0 |
|  | [57] | African Index Medicus and AJOL | Burkina Faso (Ouagadougou: University Hospital CHUO) | November 2006 - February 2008 | **Cross-sectional**  Inclusion: patients with acute gastroenteritis, HIV positive or negative  Exclusion: NR | Stool | Culture: mCCDA; Columbia Blood Agar with 5% defibrinated horse blood;  Microscopy: Gram’s stain  Biochemical tests: oxidase and catalase | 1246 | 2.3%, n=29 | 1.5%-3.3% | *C. jejuni* (1.2%, n=15)  *[C. jejuni jejuni* 0.9%, n=11  *C. jejuni doylei* 0.2%, n=3]  *C. coli* (0.3%, n=4)  *C. upsaliensis* (0.08%, n=1)  Unidentified (0.8%, n=9) |
|  | [58] | Google Scholar and AJOL | Burkina Faso (Ouagadougou: El Fateh Suka clinic and Saint Camille Medical Centre) | February, 5- March, 9, 2013 | **Cross-sectional**  Inclusion: patients with (a) or without (b) diarrhea  Exclusion: NR | Stool | Multiplex RT-PCR | 200  a.  42  b.  158 | 1%, n=2  a.  2.4%, n=1  b.  0.6%, n=1 | 0.1%-3.6%  a.  0.1%-12.6%  b.  0.0%-3.5% | *Campylobacter* spp. |
|  | [59] | Sciencedirect | Côte d’Ivoire (Dabou: Hôpital Méthodiste de Dabou and village healthcare centers) | October 2012 | **Case-control**  Inclusion: Cases: individuals aged ≥12 months with persistent diarrhea; Controls: without gastrointestinal complaints within the preceding 2 months  Exclusion: Individuals with acute diarrhea or other abdominal symptoms with a duration of <2 weeks | Stool | PCR  Luminex® GPP | 136  Cases  68  Controls  68 | 9%, n= 12  Cases  6%, n=4  Controls  12%, n=8 | 4.6%-14.9%  Cases  1.6%-14.4%  Controls  5.2%-21.9% | *Campylobacter* spp. |
|  | [60] | Google scholar | Ghana  (Kumasi, Komfo Anokye Teaching Hospital) | May - August 2013 | **Cross-sectional**  Inclusion: in-patients and out patients with gastroenteritis or urinary tract infections (UTI) from all age groups  Exclusion: NR | Stool and mid-stream urine | Culture: mCCDA with supplement;  Microscopy: Gram stain Biochemical tests: catalase and oxidase; API Campy kit tests | 202  Enteritis 128  UTI  74 | 17.3%, n=35  Enteritis  20.3%, n=26  UTI  12.2%, n=9 | 12.4%-23.3%  Enteritis  13.7%-28.3%  UTI  5.7%-21.8% | Enteritis:  *C. coli* (9.3%, n=12)  *C. jejuni* (8.6%, n=11)  [*C. jejuni* subsp. *doylei* (0.8%, n=1)]  *C. lari* (2.3%, n=3)  UTI  *C. jejuni* (5.4%, n=4)  *C. lari* (5.4%, n=4)  *C. coli* (1.4%, n=1) |
|  | [61] | PUBMED | Ghana (Asante Akim North municipality: Agogo Presbyterian Hospital (APH)) | June 2007 -October 2008 | **Case-control**  Inclusion: Cases: children ≤ 13 years with diarrhea; Control: cases without symptoms  Exclusion: Children with ongoing diarrheal disease | Stool | PCR | 1234  Cases  548  Controls  686 | 19.6%, n=242  Cases  22.6%, n=124  Controls  17.2%, n=118 | 17.4%-21.9%  Cases  19.2%-26.4%  Controls  14.5%-20.2% | *C. jejuni* |
|  | [62] | Sciencedirect | Guinea-Bissau (Bandim II and Belem communities) | January - April 2001 | **Cross-sectional**  Inclusion: children aged 4—12 years  Exclusion: NR | Stool | Culture (Medium NR) | 706 | 1.8%, n=13 | 1.0%-3.1% | *C. jejuni* (1.1%, n=8)  *C. coli* (0.4%, n=3)  Other *Campylobacter* spp. (0.3%, n=2) |
|  | [63] | PUBMED | Niger (Maradi: one hospital and 10 health centers) | April 2010 - March 2012 | **Cross-sectional**  Inclusion: Children aged 0 -59 months with watery diarrhea and signs of moderate or severe dehydration  Exclusion: NR | Stool  a. Watery diarrhea  b. Bloody diarrhea | Culture: Butzler media; microscopy: Gram stain; Motility test;  Biochemical tests: oxidase, catalase, sodium hippurate hydrolysis. | a.  3790  b.  230 | a.  11.4%, n=350  b.  8.8%, n=20 | a.  10.2%–12.6%  b.  5.7%–13.3% | *C. jejuni* |
|  | [64] | PUBMED, AJOL and Google Scholar | Nigeria (Ilorin: University of Ilorin Teaching Hospital, Private Hospitals and Primary health Centers) | December 2002 - November 2003 | **Prospective case-control**  Inclusion: Cases: child from 0-36 months old with watery, offensive diarrhea with or without blood and fever; Controls: child without diarrhea  Exclusion: NR | Rectal swabs and stool | Culture: Butzler-type medium;  Microscopy gram stain; Motility test;  Biochemical tests: oxidase and catalase, hippurate hydrolysis, H_2_S and DNA hydrolysis tests | 406  Cases  306  Controls 100 | Cases  8.2%, n=25  Controls  0%, n=0 | Cases  5.4%-11.8%  Controls  0.0%-3.6% | *C. jejuni* (4.6%, n=14)  [Biotype I (3.9%, n=12)  Biotype II (0.7%, n=2)]  *C. coli* Byotype I (3.6%, n =11) |
|  | [65] | Google Scholar | Nigeria (Sokoto State: hospitals) | NR | **Cross-sectional**  Inclusion: volunteers on admission and at outpatient clinics  Exclusion: NR | Stool | Culture: mCCDA; Microscopy: Gram stain; Biochemistry: oxidase, catalase, and H_2_S production tests | 292 | 55%, n=160 | 48.9%-60.6% | *C. jejuni* (13.0%, n=38)  *C. coli* (21.6%, n=63)  *C. lari* (20.2%, n=59) |
|  | [66] | Google Scholar | Nigeria (Orlu: Imo State University Teaching Hospital Orlu) | May 2011 - April 2014 | **Cross-sectional**  Inclusion: patients with enteric diseases (gastroenteritis and fever)  Exclusion: NR | Stool | Culture: *Campylobacter* selective medium | 988 | 18.5%, n=183 | 16.1%-21.1% | *C. jejuni* |
|  | [67] | Sciencedirect | Nigeria (Kano: Murtala Muhammad Specialist Hospital) | NR | **Prospective**  Inclusion: children with diarrhea aged 1—5 years  Exclusion: NR | Stool | Culture (medium NR) | 400 | 0.25%, n=1 | 0.0%-1.4% | *Campylobacter* spp. |
|  | [68] | PUBMED and Google Scholar | Nigeria (Lagos: Hospital?) | NR | **Case-control**  Inclusion: Cases: HIV/AIDS patients with diarrhea, Control: without diarrhea  Exclusion: NR | Stool and rectal swabs | NR | 160  Cases  84  Controls  76 | 5%, n= 8  Cases  7%, n=6  Controls  2.5%, n=2 | 2.2%-9.6%  Cases  2.7%-14.9%  Control  0.3%-9.2% | *Campylobacter* spp. |
|  | [69] | Google Scholar | Nigeria (Zuru Kebbi State: pig rearing areas) | September, 2013 - February, 2014 | **Cross-sectional**  Inclusion: volunteer pig farmers and their household members  Exclusion: NR | Stool | Culture: CCDA; Microscopy: Gram stain; Biochemical tests catalase, oxidase, hippurate hydrolysis and H_2_S production | 150 | 62.7%, n=94 | 54.4%-70.4% | *C. coli* (38.0%, n=57)  *C. jejuni* (15.3%, n=23)  *C. upsaliensis* (4.7%, n=7)  *C. hyointestinalis* (3.3%, n=5)  *C. lari* (1.3%, n=2) |
|  | [70] | PUBMED and Google Scholar | Nigeria (Ile-Ife: Ife State Hospital and Primary Health Centre) | April - December 1997 | **Prospective case-control**  Inclusion: Cases: patients with diarrhea; Controls: patients without diarrhea for the past 2 weeks (children in both groups_  Exclusion: NR | Stool and rectal swabs | Culture: Butzler-type medium  Microscopy: Gram stain; Motility test; Biochemical tests: oxidase, catalase. | 403  Cases  303  Controls  100 | 15.9%, n=64  Cases  19.1%, n=58  Controls  6%, n=6 | 12.5%-19.8%  Cases  14.9%-24.0%  Controls  2.2%-12.6% | *C. jejuni* (n= 14)  [Biotype I (n=10)  Biotype II (n= 4)]  *C. coli* Biotype I (n= 16)  *it is not known to which group the isolates belong. |
|  | [71] | PUBMED | Nigeria (Enugu State: University of Nigeria Teaching Hospital) | 2002-2006 | **Cross-sectional**  Inclusion: children with diarrhea < 5 years  Exclusion: NR | Stool | Culture: Butzler-type medium [Brucella agar and 10% defibrinated sheep blood with supplement, SR85])  Other methods (NR) | 514 | 8.3%, n=43 | 6.1%-11.1% | *C. jejuni* (7.8%?, n=40)  Other *Campylobacter* spp. |
|  | [72] | Google Scholar | Nigeria (Kaduna State, Zaria: Banzazzau Comprehensive Health Centre and Gambo Suwaba  General Hospital) | NR | **Case-control**  Inclusion: Cases: Stools from children ≤ 3 years with diarrhea; Controls: matched by  age and gender attending the clinics, without diarrhea; Stool samples collected in appropriate clean, dry, wide-mouthed and leak proof container.  Exclusion: Stool sample of more than 2 hours duration between passage and submission to the laboratory; sample from patients known to have recently been on any antibiotic within the past two weeks; samples contaminated with urine, water or any substance; samples submitted in inappropriate containers. | Stool or rectal swabs | Culture: Butzler-type selective medium; subculture onto blood agar;  Microscopy: Gram stain; Motility test;  Biochemical tests: oxidase, catalase, hippurate hydrolysis test, (H_2_S) test and DNA hydrolysis | 361  Cases  261 Controls 100 | Cases  15.3%, n= 40  Controls  0%, n=0 | Cases  11.2% -20.3%  Controls  0.0%- 3.6% | *C. jejuni* (11.1%, n=29)  [Biotype I (5.7%, n=15)  Biotype II (2.3%, n=6)  Biotype III (1.5%, n=4)  Biotype IV (1.5%, n=4)]  *C. coli* (4.2%, n= 11)  [Biotype I (2.3%, n=6)  Biotype II (1.9%, n=5)] |
|  | [73] | Google Scholar | Nigeria (Jos University  Teaching Hospital) | September 2012 - March 2013 | **Case-control**  Inclusion: Cases: < 5 years with diarrhea, Nnot on antibiotics two week before inclusion into the study; Controls: without diarrhea.  Exclusion: > 5 years; samples delayed for more than 2h after collection; dry swab specimens. | Stool and rectal swabs | Campy-BAP selective agar plate; Microscopy: Gram stain; Biochemical tests: oxidase test, hippurate hydrolysis, H_2_S production DNA hydrolysis | 360  Cases  260  Controls  100 | Cases  6.5%, n= 17  Controls  2.0%, n=2 | Cases  3.9%-10.3%  Controls  0.2%-7.0% | Cases  *C. jejuni* (4.2%, n= 11)  *C. coli* (1.9%, n= 5)  *C. lari* (0.4%, n=1) |
|  | [74] | Google Scholar | Nigeria (Ibadan: Institute of Advanced Medical Research and Training (IMRAT), University College Hospital (UCH)) | February - March  2017 | **Cross-sectional**  Inclusion: HIV infected patients  Exclusion**:** NR | Stool | Culture: Nutrient agar and CCDA with supplement; Microscopy: Gram stain; Motility test; Biochemical tests: oxidase, catalase, sulphide reduction, hippurate hydrolysis, sugar fermentation;  Susceptibility to nalidixic  acid and cephalothin | 100 | 68%, n=68 | 57.9%-77.0% | *C. jejuni* (30%, n=30)  *C. coli* (20%, n=20)  *C. lari* (23%, n=23)  *C. fetus* (15%, n=15)  *C. upsaliensis* (32%, n=32) |
|  | [75] | Google Scholar | Nigeria (Kaduna State, 3 senatorial districts: 5 hospitals) | August – December 2017 | **Cross-sectional**  Inclusion: HIV infected patients ≥ 10 years with diarrhea | Stool | Culture: *Campylobacter*  sheep blood agar, mCCDA with supplement  Biochemical tests: Catalase, Oxidase, Nitrate reduction; API Campy kits tests;  PCR | 230 | 19.6%, n=45 | 14.6%-25.3% | *C. jejuni* (6.5%, n=15)  *C. coli* (8.7%, n=20)  *C. fetus* (3.0%, n=7)  *C. hyointestinalis* (1.3%, n=3) |
|  | [76] | PUBMED | Senegal (Dakar: Saint-Martin dispensary – primary health care organization) | September 2007 - March 2008 | **Cross-sectional**  Inclusion: children aged 1 month to 5 years with acute diarrhea  Exclusion: NR | Stool | Culture: Preston broth medium and subculture onto Karmali selective agar | 176 | 3%, n=6 | 1.3%-7.3% | *C. jejuni* |
|  | [77] | Sciencedirect | Senegal (Dakar: Hôpital Principal and Fann Teaching Hospital) | NR | **Case-control**  Inclusion: Cases: HIV-infected patients with diarrhea (HIV+D+), HIV seronegative patients with diarrhea (HIV- D+) who had not received antibiotics during the previous 2 weeks; Controls: HIV-infected patients without diarrhea (HIV+ D-); HIV  seronegative controls without diarrhea (HIV- D-).  Exclusion: NR | Stool | Standard methods (not specified) | 594  Cases  HIV+D+  158  HIV-D+  121  Controls  HIV+D-  160  HIV-D-  155 | 0.5%, n=3  Cases  HIV+D+  0.6%, n=1  HIV-D+  1.6%, n=2  Controls  HIV+D-  0%, n=0  HIV-D-  0%, n=0 | 0.1%-1.47%  Cases  HIV+D+  0.0%-3.5%  HIV-D+  0.2%-5.9%  Controls  HIV+D-  0.0%-2.3%  HIV-D-  0.0%- 2.4% | *C. fetus* (0.6%, n=1) HIV+D+  *C. jejuni* (1.6%, n=2) HIV-D+ |

NR – Not Reported

**References**

1. Beyene G, Haile-Amlak A. Antimicrobial sensitivity pattern of *Campylobacter* species among children in Jimma University Specialized Hospital, Southwest Ethiopia. Ethiop J Heal Dev. 2004;18: 185–189. doi:10.4314/ejhd.v18i3.9958

2. Mitike G, Kassu A, Genetu A, Nigussie D. *Campylobacter* enteritis among children in Dembia District, Northwest Ethiopia. East Afr Med J. 2000;77: 654–657. doi:10.4314/eamj.v77i12.46764

3. Tafa B, Sewunet T, Tassew H, Asrat D. Isolation and Antimicrobial Susceptibility Patterns of *Campylobacter* Species among Diarrheic Children at Jimma, Ethiopia. Int J Bacteriol. 2014;2014: 1–7. doi:10.1155/2014/560617

4. Ewnetu D, Muhret A. Prevalence and Antimicrobial Resistance of *Campylobacter* Isolates from Humans and Chickens in Bahir Dar, Ethiopia. Foodborne Pathog Dis. 2010;7: 667–670. doi:10.1089/fpd.2009.0433

5. Mulatu G, Getenet B, Ahmed Z. Prevalence of Shigella, Salmonella and *Campylobacter* species and their susceptibility patters among under five children with diarrhea in Hawassa Town, South Ethiopia. Ethiop J Heal Sci. 2014;24: 101–108. doi:10.4314/ejhs.v24i2.1

6. Lengerh A, Moges F, Unakal C, Anagaw B. Prevalence, associated risk factors and antimicrobial susceptibility pattern of *Campylobacter* species among under five diarrheic children at Gondar University Hospital, Northwest Ethiopia. BMC Pediatr. 2013;13. doi:10.1186/1471-2431-13-82

7. Kebede A, Aragie S, Shimelis T. The common enteric bacterial pathogens and their antimicrobial susceptibility pattern among HIV-infected individuals attending the antiretroviral therapy clinic of Hawassa university hospital, southern Ethiopia. Antimicrob Resist Infect Control. 2017;6: 1–7. doi:10.1186/s13756-017-0288-7

8. Shapiro RL, Kumar L, Phillips-Howard P, Wells JG, Adcock P, Brooks J, et al. Antimicrobial‐Resistant Bacterial Diarrhea in Rural Western Kenya. J Infect Dis. 2001;183: 1701–1704. doi:10.1086/320710

9. Mogeni DO, Otieno CL, Awiti G, Wamola N, Fields B, Neatherlin J, et al. Detection of viral respiratory and gastrointestinal pathogens among healthy adults and children of an informal settlement (Kibera) in Nairobi, Kenya. Int J Infect Dis. 2014;21: 223. doi:10.1016/j.ijid.2014.03.885

10. van Eijk A., Brooks JT, Adcock PM, Garrett V, Eberhard M, Rosen DH, et al. Diarrhea in children less than two years of age with known HIV status in Kisumu, Kenya. Int J Infect Dis. 2010;14: e220–e225. doi:10.1016/j.ijid.2009.06.001

11. Brooks JT, Shapiro RL, Kumar L, Wells JO, Phillips-Howard PA, Shi Y-P, et al. Epidemiology of sporadic bloody diarrhea in Rural Western Kenya. Am J Trop Med Hyg. 2003;68: 671–677.

12. Pavlinac PB, John-Stewart GC, Naulikha JM, Onchiri FM, Denno DM, Odundo EA, et al. High-Risk Enteric Pathogens Associated with HIV-Infection and HIV-Exposure in Kenyan Children with Acute Diarrhea. AIDS. 2014;28: 2287–2296. doi:10.1097/QAD.0000000000000396

13. Tickell KD, Pavlinac PB, John-Stewart GC, Denno DM, Richardson BA, Naulikha JM, et al. Impact of Childhood Nutritional Status on Pathogen Prevalence and Severity of Acute Diarrhea. Am J Trop Med Hyg. 2017;97: 1337–1344. doi:10.4269/ajtmh.17-0139

14. Ongwae ZH, Mwamburi LA, Kakai R. Multiple drug resistance of *Campylobacter jejuni* and *Shigella* isolated from diarrhoeic children aged under five years admitted at Kapsabet County Hospital, Kenya. Proceedings of the 2018 International Women in Science Without Borders (WISWB) – Indaba. Johannesburg, South Africa; 2018. pp. 1–3.

15. O’Reilly CE, Jaron P, Ochieng B, Nyaguara A, Tate JE, Parsons MB, et al. Risk Factors for Death among Children Less than 5 Years Old Hospitalized with Diarrhea in Rural Western Kenya , 2005–2007: A Cohort Study. PLoS Med. 2012;9: 2005–2007. doi:10.1371/journal.pmed.1001256

16. Beatty ME, Ochieng JB, Chege W, Kumar L, Okoth G, Shapiro RL, et al. Sporadic paediatric diarrhoeal illness in urban and rural sites in Nyanza province, Kenya. East Afr Med J. 2009;86: 387–398.

17. Brooks JT, Ochieng JB, Kumar L, Okoth G, Shapiro RL, Wells JG, et al. Surveillance for Bacterial Diarrhea and Antimicrobial Resistance in Rural Western Kenya, 1997–2003. Clin Infect Dis. 2006;43: 383–401. doi:10.1086/505866

18. Swierczewski BE, Odundo EA, Koech MC, Ndonye JN, Kirera RK, Odhiambo CP, et al. Surveillance for enteric pathogens in a case-control study of acute diarrhea in Western Kenya. Trans R Soc Trop Med Hyg. 2013;107: 83–90. doi:10.1093/trstmh/trs022

19. Conan A, O’Reilly CE, Ogola E, Ochieng JB, Blackstock AJ, Omore R, et al. Animal-related factors associated with moderate-to-severe diarrhea in children younger than five years in western Kenya: A matched case-control study. PLoS Negl Trop Dis. 2017;11: e0005795. doi:10.1371/journal.pntd.0005795

20. Gitahi N, Gathura PB, Gicheru MM, Wandia BM, Nordin A. Multidrug-resistant Campylobacter jejuni, *Campylobacter coli* and *Campylobacter lari* isolated from asymptomatic school-going children in Kibera slum, Kenya [version 2; peer review: 1 approved, 1 approved with reservations]. F1000Research. 2020;9. doi:10.12688/f1000research.21299.2

21. Randremanana RV, Randrianirina F, Sabatier P, Rakotonirina HC, Randriamanantena A, Razanajatovo IM, et al. *Campylobacter* infection in a cohort of rural children in Moramanga, Madagascar. BMC Infect Dis. 2014;14. doi:10.1186/1471-2334-14-372

22. Randremanana RV, Razafindratsimandresy R, Andriatahina T, Randriamanantena A, Ravelomanana L, Randrianirina F, et al. Etiologies, Risk Factors and Impact of Severe Diarrhea in the Under-Fives in Moramanga. PLoS One. 2016;11: e0158862. doi:10.1371/journal.pone.0158862

23. Randremanana RV. Impacts de l’environnement sur les diarrhées infantiles à Madagascar: Analyse du risque *Campylobacter*. Université de Grenoble. 2013. Available: https://tel.archives-ouvertes.fr/tel-00872059/document

24. Mason J, Iturriza-Gomara M, O’Brien SJ, Ngwira BM, Dove W, Maiden MCJ, et al. *Campylobacter* Infection in Children in Malawi Is Common and Is Frequently Associated with Enteric Virus Co-Infections. PLoS One. 2013;8: e59663. doi:10.1371/journal.pone.0059663

25. Versloot CJ, Attia S, Bourdon C, Richardson SE, Potani I, Bandsma RHJ, et al. Intestinal pathogen clearance in children with severe acute malnutrition is unrelated to inpatient morbidity. Clin Nutr ESPEN. 2018;24: 109–113. doi:10.1016/j.clnesp.2018.01.004

26. Attia S, Versloot CJ, Voskuijl W, Vliet SJV, Giovanni VD, Zhang L, et al. Mortality in children with complicated severe acute malnutrition is related to intestinal and systemic inflammation: an observational cohort study. Am J Clin Nutr. 2016;104: 1441–1449. doi:10.3945/ajcn.116.130518

27. Mandomando IM, Macete EV, Ruiz J, Sanz S, Abacassamo F, Vallès X, et al. Etiology of diarrhea in children younger than 5 years of age admitted in a rural hospital of Southern Mozambique. Am J Trop Med Hyg. 2007;76: 522–527.

28. Knee J, Sumner T, Adriano Z, Berendes D, Bruijn E, Schmidt W-P, et al. Risk factors for childhood enteric infection in urban Maputo, Mozambique: A cross-sectional study. PLoS Negl Trop Dis. 2018;12: e0006956. doi:10.1371/journal.pntd.0006956

29. Nhampossa T, Mandomando I, Acacio S, Quintó L, Vubil D, Ruiz J, et al. Diarrheal Disease in Rural Mozambique: Burden, Risk Factors and Etiology of Diarrheal Disease among Children aged 0–59 Months Seeking Care at Health Facilities. PLoS One. 2015;10: e0119824. doi:10.1371/journal.pone.0119824

30. Kabayiza J-C, Andersson ME, Nilsson S, Baribwira C, Muhirwa G, Bergström T, et al. Diarrhoeagenic microbes by real-time PCR in Rwandan children under 5 years of age with acute gastroenteritis. Clin Microbiol Infect. 2014;20: O1128–O1135. doi:10.1111/1469-0691.12698

31. Kabayiza J-C, Andersson ME, Nilsson S, Bergström T, Muhirwa G, Lingh M. Real-time PCR Identification of Agents Causing Diarrhea in Rwandan Children Less Than 5 Years of Age. Pediatr Infect Dis J. 2014;33: 1037–1042. doi:10.1097/INF.0000000000000448

32. Mshana SE, Joloba M, Kakooza A, Kaddu-Mulindwa D. *Campylobacter* spp among Children with acute diarrhea attending Mulago hospital in Kampala - Uganda. Afr Heal Sci. 2009;9: 201–205.

33. Chuma IS, Nonga HE, Mdegela RH, Kazwala RR. Epidemiology and RAPD-PCR typing of thermophilic campylobacters from children under five years and chickens in Morogoro Municipality, Tanzania. BMC Infect Dis. 2016;16. doi:10.1186/s12879-016-2031-z

34. Gosselin KB, Aboud S, McDonald CM, Moyo S, Khavari N, Manji K, et al. Etiology of Diarrhea, Nutritional Outcomes, and Novel Intestinal Biomarkers in Tanzanian infants. JPGN. 2017;64: 104–108. doi:10.1097/MPG.0000000000001323

35. Oketcho R, Nyaruhucha CNM, Taybali S, Karimuribo ED. Influence of enteric bacteria and parasite infection and nutritional status on diarrhoea occurrence in six to 60 month old children admitted at a Regional Hospital in Morogoro, Tanzania. Tanzan J Heal Res. 2012;14: 1–15. doi:10.4314/thrb.v14i2.3

36. Deogratias A-P, Mushi MF, Paterno L, Tappe D, Seni J, Kabymera R, et al. Prevalence and determinants of *Campylobacter* infection among under five children with acute watery diarrhea in Mwanza, North Tanzania. Arch Public Heal. 2014;72. doi:10.1186/2049-3258-72-17

37. Kusiluka LJM, Karimuribo ED, Mdegela RH, Luoga EJ, Munishi PKT, Mlozi MRS, et al. Prevalence and impact of water-borne zoonotic pathogens in water, cattle and humans in selected villages in Dodoma Rural and Bagamoyo districts, Tanzania. Phys Chem Earth. 2005;30: 818–825. doi:10.1016/j.pce.2005.08.025

38. Mdegela RH, Nonga HE, Ngowi HA, Kazwala RR. Prevalence of Thermophilic *Campylobacter* Infections in Humans, Chickens and Crows in Morogoro, Tanzania. J Vet Med B Infect Dis Vet Public Heal. 2006;53: 116–121. doi:10.1111/j.1439-0450.2006.00926.x.

39. Komba EVG, Mdegela RH, Msoffe PLM, Nielsen LN, Ingmer H. Prevalence, Antimicrobial Resistance and Risk Factors for Thermophilic *Campylobacter* Infections in Symptomatic and Asymptomatic Humans in Tanzania. Zoonoses Public Heal. 2015;62: 557–568. doi:10.1111/zph.12185

40. Elfving K, Andersson M, Msellem MI, Welinder-Olsson C, Petzold M, Björkman A, et al. Real-Time PCR Threshold Cycle Cutoffs Help To Identify Agents Causing Acute Childhood Diarrhea in Zanzibar. J Clin Microbiol. 2014;52: 916–923. doi:10.1128/JCM.02697-13

41. Andersson ME, Elfving K, Shakely D, Nilsson S, Msellem M, Trollfors B, et al. Rapid Clearance and Frequent Reinfection With Enteric Pathogens Among Children With Acute Diarrhea in Zanzibar. Clin Infect Dis. 2017;65: 1371–1377. doi:10.1093/cid/cix500

42. Chiyangi H, Muma J., Malama S, Manyahi J, Abade A, Kwenda G, et al. Identification and antimicrobial resistance patterns of bacterial enteropathogens from children aged 0–59 months at the University Teaching Hospital, Lusaka, Zambia: a prospective cross sectional study. BMC Infect Dis. 2017;17. doi:10.1186/s12879-017-2232-0

43. Gwavava C, Chihota VN, Gangaidzo IT, Gumbo T. Dysentery in patients infected with human immunodeficiency virus in Zimbabwe: an emerging role for *Schistosoma mansoni* and *Escherichia coli* O157? Ann Trop Med Parasitol. 2001;95: 509–513. doi:10.1080/00034980120076235

44. Pelkonen T, Dias M, Roine I, Anjos E, Freitas C, Peltola H, et al. Potential Diarrheal Pathogens Common Also in Healthy Children in Angola. Pediatr Infect Dis J. 2018;37: 424–428. doi:10.1097/INF.0000000000001781

45. Zash RM, Shapiro RL, Leidner J, Wester C, McAdam AJ, Hodinka RL, et al. The aetiology of diarrhoea, pneumonia and respiratory colonization of HIV-exposed infants randomized to breast- or formula-feeding. Paediatr Int Child Heal. 2016;36: 189–197. doi:10.1179/2046905515Y.0000000038

46. Rowe JS, Shah SS, Motlhagodi S, Bafana M, Tawanana E, Truong HT, et al. An Epidemiologic Review of Enteropathogens in Gaborone, Botswana: Shifting Patterns of Resistance in an HIV Endemic Region. PLoS One. 2010;5: e10924. doi:10.1371/journal.pone.0010924

47. Pernica JM, Steenhoff AP, Welch H, Mokomane M, Quaye I, Arscott-Mills T, et al. Correlation of Clinical Outcomes With Multiplex Molecular Testing of Stool From Children Admitted to Hospital With Gastroenteritis in Botswana. J Pediatr Infect Dis Soc. 2015;5: 312–318. doi:10.1093/jpids/piv028

48. Alam K, Lastovica AJ, Le Roux E, Hossain MA, Islam MN, Sen SK, et al. Clinical Characteristics and Serotype Distribution of *Campylobacter jejuni* and *Campylobacter coli* Isolated from Diarrhoeic Patients in Dhaka, Bangladesh, and Cape Town, South Africa. Bangladesh J Microbiol. 2006;23: 121–124. doi:10.3329/bjm.v23i2.875

49. Thobela MS, Smith AM, Moonsamy S, du Plessis H, Govender N, Keddy KH. Detection of *Campylobacter* species in stool specimens from patients with symptoms of acute flaccid paralysis in South Africa. J Infect Dev Ctries. 2018;12: 542–549. doi:10.3855/jidc.9795

50. Obi CL, Bessong PO. Diarrhoeagenic bacterial pathogens in HIV-positive patients with diarrhoea in rural communities of Limpopo Province, South Africa. J Heal Popul Nutr. 2002;20: 230–234.

51. Samie A, Obi CL, Barrett LJ, Powell SM, Guerrant RL. Prevalence of *Campylobacter* species, *Helicobacter pylori* and *Arcobacter* species in stool samples from the Venda region, Limpopo, South Africa: Studies using molecular diagnostic methods. J Infect. 2007;54: 558–566. doi:10.1016/j.jinf.2006.10.047

52. Kullin B, Meggersee R, D’Alton J, Galvão B, Rajabally N, Whitelaw A, et al. Prevalence of gastrointestinal pathogenic bacteria in patients with diarrhoea attending Groote Schuur Hospital, Cape Town, South Africa. S Afr Med J. 2015;105: 121–125. doi:10.7196/SAMJ.8654

53. Samie A, Guerrant RL, Barrett L, Bessong PO, Igumbor EO, Obi CL. Prevalence of Intestinal Parasitic and Bacterial Pathogens in Diarrhoeal and Non-diarroeal Human Stools from Vhembe District, South Africa. J Heal Popul Nut. 2009;27: 739–745. doi:10.3329/jhpn.v27i6.4325

54. Samie A, Ramalivhana J, Igumbor EO, Obi CL. Prevalence, Haemolytic and Haemagglutination Activities and Antibiotic Susceptibility Profiles of *Campylobacter* spp. Isolated from Human Diarrhoeal Stools in Vhembe District, South Africa. J Heal Popul Nutr. 2007;25: 406–413.

55. Kalule JB, Smith AM, Vulindhlu M, Tau NP, Nicol MP, Keddy KH, et al. Prevalence and antibiotic susceptibility patterns of enteric bacterial pathogens in human and non-human sources in an urban informal settlement in Cape Town, South Africa. BMC Microbiol. 2019;19. doi:10.1186/s12866-019-1620-6

56. Bonkoungou IJO, Haukka K, Österblad M, Hakanen AJ, Traoré AS, Barro N, et al. Bacterial and viral etiology of childhood diarrhea in Ouagadougou, Burkina Faso. BMC Pediatr. 2013;13. doi:10.1186/1471-2431-13-36

57. Sangaré L, Nikiéma AK, Zimmermann S, Sanou I, Congo-Ouédraogo M, Diabaté A, et al. *Campylobacter* spp. epidemiology and antimicrobial susceptibility in a developing country, Burkina Faso (West Africa). Arf J Cln Exper Microbiol. 2012;13: 106–111. doi:10.4314/ajcem.v13i2.9

58. Sawadogo S, Diarra B, Bisseye C, Compaore TR, Djigma FW, Ouermi D, et al. Molecular Diagnosis of *Shigella*, *Salmonella* and *Campylobacter* by Multiplex Real-Time PCR in Stool Culture Samples in Ouagadougou (Burkina Faso). Sudan J Med Sci. 2017;12: 163–173. doi:10.18502/sjms.v12i3.931

59. Becker SL, Chatigre JK, Gohou J-P, Coulibaly JT, Leuppi R, Polman K, et al. Combined stool-based multiplex PCR and microscopy for enhanced pathogen detection in patients with persistent diarrhoea and asymptomatic controls from Côte d’Ivoire. Clin Microbiol Infect. 2015;21: 591.e1-591.e10. doi:10.1016/j.cmi.2015.02.016

60. Karikari AB, Obiri-Danso K, Frimpong EH, Krogfelt KA. Antibiotic Resistance in *Campylobacter* Isolated from Patients with Gastroenteritis in a Teaching Hospital in Ghana. Open J Med Microbiol. 2017;7: 1–11. doi:10.4236/ojmm.2017.71001

61. Krumkamp R, Sarpong N, Schwarz NG, Adelkofer J, Loag W, Eibach D, et al. Gastrointestinal Infections and Diarrheal Disease in Ghanaian Infants and Children: An Outpatient Case-Control Study. PLoS Negl Trop Dis. 2015;9: e0003568. doi:10.1371/journal.pntd.0003568

62. Steenhard NR, Ørnbjerg N, Mølbak K. Concurrent infections and socioeconomic determinants of geohelminth infection: a community study of schoolchildren in periurban Guinea-Bissau. Trans R Soc Trop Med Hyg. 2009;103: 839–845. doi:10.1016/j.trstmh.2009.05.005

63. Langendorf C, Hello SL, Moumouni A, Gouali M, Mamaty A-A, Grais RF, et al. Enteric Bacterial Pathogens in Children with Diarrhea in Niger: Diversity and Antimicrobial Resistance. PLoS One. 2015;10: e0120275. doi:10.1371/journal.pone.0120275

64. Samuel SO, Aboderin AO, Akanbi AA, Adegboro B, Smith SI, Coker AO. *Campylobacter* enteritis in Ilorin, Nigeria. East Afr Med J. 2006;83: 478–484. doi:10.4314/eamj.v83i09.46770

65. Nwankwo IO, Faleke OO, Salihu MD, Magaji AA, Musa U, Garba J, et al. Detection and viability of *Campylobacter* species isolates from different species of poultry and humans in Sokoto State, Nigeria. Int J One Heal. 2016;2: 19–23. doi:10.14202/ijoh.2016.19-23

66. Obiajuru IOC, Anoule FC, Adogu POU. Emergence of Campylobacteriosis in Orlu, Imo State South Eastern Nigeria, and its Antibiotic Susceptibility. Carib J Sci Tech. 2015;3: 798–804.

67. Hassan-Hanga F, Osisuni K, Ibrahim M. Infectious Diseases in Under-five Children in Kano, North western Nigeria. Int J Infect Dis. 2008;12: e83. doi:10.1016/j.ijid.2008.05.206

68. Smith SI, Otuonye MN, Omonigbehin EA, Nkoth A, Okany CC, Ariyo F, et al. Prevalence of *Campylobacter* species among HIV/AIDS patients in Nigeria. Br J Biomed Sci. 2002;59: 162–163. doi:10.1080/09674845.2002.11978035

69. Gwimi PB, Faleke OO, Salihu MD, Magaji AA, Abubakar MB, Nwankwo IO, et al. Prevalence of *Campylobacter* species in fecal samples of pigs and humans from Zuru Kebbi State, Nigeria. Int J One Heal. 2015;1: 1–5.

70. Aboderin AO, Smith SI, Oyelese AO, Onipede AO, Zailani SB, Coker AO. Role of *Campylobacter jejuni/coli* in diarrhoea in Ile-Ife, Nigeria. East Afr Med J. 2002;79: 423–426.

71. Ohanu ME, Offune J. The Prevalence of *Campylobacter* in Childhood Diarrhoea in Enugu State of Nigeria. J Commun Dis. 2009;41: 117–120.

72. Ibrahim A. *Campylobacter* enteritis in Zaria, North Western Nigeria. Ahmadu Bello University Teaching Hospital. 2014. Available: https://dissertation.npmcn.edu.ng/index.php/FMCPath/article/view/1366

73. Udoh UA. Characterization of *Campylobacter jejuni* as an enteric pathogen in under five children in Jos, Nigeria. Jos University Teaching Hospital. 2013. Available: https://dissertation.npmcn.edu.ng/index.php/FMCPath/article/view/1193/1388

74. Adedapo AE. Prevalence and drug resistance patterns of *Campylobacter* and *Listeria* species from the stool samples of HIV patients in Ibadan, Nigeria. University of Ibadan. 2018.

75. Ogbomon EO, Whong CMZ, Doko MHI, Magaji SN, Addai TI, Orukotan YF. Prevalence of *Campylobacter* spp. among diarrhoeic HIV-patients in Kaduna, Nigeria. IJAMBR. 2019;7: 70–78. doi:10.33500/ijambr.2019.07.009

76. Sire J-M, Garin B, Chartier L, Fall NK, Tall A, Seck A, et al. Community-acquired infectious diarrhoea in children under 5 years of age in Dakar, Senegal. Paediatr Int Child Heal. 2013;33: 139–144. doi:10.1179/2046905512Y.0000000046

77. Gassama A, Sow PS, Fall F, Camara P, Philippe H, Guyèye-N’diaye A, et al. Ordinary and opportunistic enteropathogens associated with diarrhea in senegalese adults in relation to Human Immunodeficiency Virus serostatus. Int J Infect Dis. 2001;5: 192–198. doi:10.1016/S1201-9712(01)90069-4
